# A Simple Structure Conjugated Polymer for High Mobility Organic Thin Film Transistors Processed from Nonchlorinated Solvent

**DOI:** 10.1002/advs.201902412

**Published:** 2019-10-29

**Authors:** Zhongli Wang, Xianneng Song, Yu Jiang, Jidong Zhang, Xi Yu, Yunfeng Deng, Yang Han, Wenping Hu, Yanhou Geng

**Affiliations:** ^1^ School of Materials Science and Engineering and Tianjin Key Laboratory of Molecular Optoelectronic Science Tianjin University Tianjin 300072 P. R. China; ^2^ Collaborative Innovation Center of Chemical Science and Engineering (Tianjin) Tianjin 300072 P. R. China; ^3^ School of Science and Tianjin Key Laboratory of Molecular Optoelectronic Science Tianjin University Tianjin 300072 P. R. China; ^4^ Joint School of National University of Singapore and Tianjin University International Campus of Tianjin University Binhai New City Fuzhou 350207 P. R. China; ^5^ State Key Laboratory of Polymer Physics and Chemistry Chinese Academy of Sciences Changchun 130022 P. R. China

**Keywords:** donor–acceptor conjugated polymers, hole mobility, nonchlorinated solvents, organic thin film transistors, side chains

## Abstract

Herein, a simple structure, nonchlorinated solvent processable high mobility donor–acceptor conjugated polymer, poly(2,5‐bis(4‐hexyldodecyl)‐2,5‐dihydro‐3,6‐di‐2‐thienyl‐pyrrolo[3,4‐*c*]pyrrole‐1,4‐dione‐alt‐thiophene) (PDPPT3‐HDO), is reported. The enhanced solubility in nonchlorinated solvent is realized based on a denser alkyl side chains strategy by incorporating small size comonomer thiophene. An associated benefit of thiophene comonomer is the remarkable structural simplicity of the resulting polymer, which is advantageous for industrial scaling up. The alkyl side chain density and structure of PDPPT3‐HDO can efficiently control the self‐assembly properties in solution and film. By bar coating from *o*‐xylene solution, PDPPT3‐HDO forms aligned films and exhibits high hole mobility of up to 9.24 cm^2^ V^−1^ s^−1^ in organic thin film transistors (OTFTs). Notably, the bar‐coated OTFT based on PDPPT3‐HDO shows a close to ideal transistor model and a high mobility reliability factor of 87%. The multiple benefits of increased side chain density strategy may encourage the design of high mobility polymers that meet the requirements of mass production of OTFT materials and devices.

## Graphical abstract

A simple structure, nonchlorinated solvent processable donor–acceptor conjugated polymer based on a denser alkyl side chains strategy is obtained and exhibits reliable high hole mobility of up to 9.24 cm^2^ V^−1^ s^−1^ in organic thin film transistor (OTFT) using a bar coating technique, which has great potential for large‐scale material and device manufacturing of high mobility OTFTs.

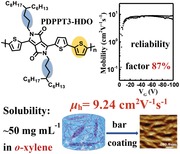

High mobility conjugated polymers (CPs) for organic thin film transistors (OTFTs) have attracted tremendous academic and industrial attention due to their excellent solution processability and mechanical properties.^1^, ^2^, ^3^, ^4^ In recent years, the emergence of donor–acceptor (D‐A) CPs has boosted the mobility of CP‐based OTFTs to over 10 cm^2^ V^−1^ s^−1^.^5^, ^6^, ^7^, ^8^, ^9^, ^10^, ^11^ However, most of these OTFTs with “record‐high mobility” are fabricated with harmful chlorinated solvents to ensure solubility or optimize device performance. Replacing these toxic chlorinated solvents with environmentally benign ones is of priority for realizing commercial solution processing of OTFTs.^12^, ^13^ In addition, simple chemical structure and high throughput film deposition technique are also prerequisites for industrial scale productivity due to economy reasons.^14^, ^15^

The solubility of CPs is generally endowed by solubilizing alkyl side chains to form hairy rod structures. The alkyl side chains adopt either end‐to‐end or interdigitated arrangement and allow subtle regulation of self‐assembly of the polymers in solution and film.^16^ In most cases, a high head‐to‐tail regioregularity of the conjugated backbones is preferred to obtain high crystallinity for enhanced charge transport.^16^, ^17^, ^18^ The high crystallinity of the conjugated backbones nevertheless prohibits efficient solute–solvent intermolecular interactions thus poor solubility is often encountered in nonchlorinated solvents.^19^, ^20^

A common method to increase solubility in nonchlorinated solvent is to reduce regioregularity by random copolymerization of asymmetric monomers or more than two monomers.^21^, ^22^, ^23^, ^24^ Another method is to introduce solubilizing side chains other than alkyls to increase solute–solvent compatibility.^25^, ^26^, ^27^ However, regioirregularity of the backbones may compromise on charge transport performance due to less ordered microstructures. At the same time, asymmetric monomers or non‐alkyl side chains are often related to time‐consuming synthesis and at the cost of device performance as well. Accordingly, nonchlorinated solvent processed OTFTs with reliable mobility approaching 10 cm^2^ V^−1^ s^−1^ has not been reported yet (**Figure** 1a and Table S1 in the Supporting Information). Given the above concerns, developing CPs that can be facilely synthesized and processed with environmentally benign solvents while maintaining high charge carrier mobilities in OTFTs is especially urgent.

**Figure 1:**
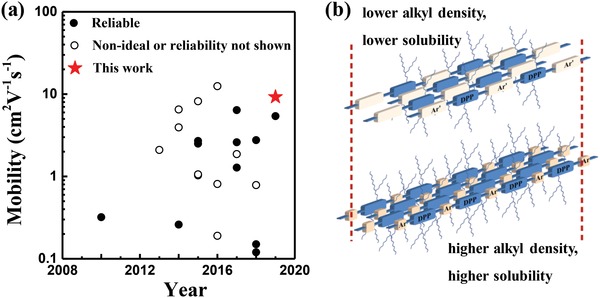
a) The development of OTFTs processed from nonchlorinated solvents in recent 10 years. b) Schematic diagram of structural design of increasing alkyl chain density by copolymerization of dithienyl‐DPP (DPP) units with small size comonomer (Ar) instead of larger aromatic comonomers (Ar').

Herein, we synthesized a diketopyrrolopyrrole (DPP)‐based polymer poly(2,5‐bis(4‐hexyldodecyl)‐2,5‐dihydro‐3,6‐di‐2‐thienyl‐pyrrolo[3,4‐*c*]pyrrole‐1,4‐dione‐alt‐thiophene) (PDPPT3‐HDO) (**Scheme** 1), and found that simply increasing alkyl side chain density by using small comonomer is sufficient to obtain D‐A CPs soluble in nonchlorinated solvent. The polymer can form aligned films featuring well‐ordered fiber‐like morphology by bar coating from *o*‐xylene. With these films, OTFTs of mobility up to 9.24 cm^2^ V^−1^ s^−1^ have been achieved. Most importantly, this value is highly reliable with a reliability factor of 87%. To the best of our knowledge, this is the highest mobility obtained from nonchlorinated solvent processed OTFTs when mobility reliability is considered (Figure 1a). The facile synthesis and good processability in nonchlorinated solvent along with high device performance make this polymer a potential candidate for future application.

**Scheme 1:**
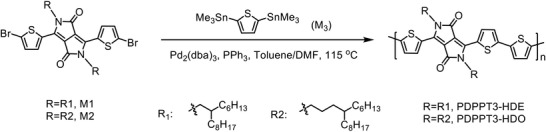
Chemical structures and synthesis of the polymers.

The structures of PDPPT3‐HDO and a reference polymer poly(2,5‐bis(2‐hexyldecyl)‐2,5‐dihydro‐3,6‐di‐2‐thienyl‐pyrrolo[3,4‐*c*]pyrrole‐1,4‐dione‐alt‐thiophene) (PDPPT3‐HDE)^28^ and related synthesis are shown in Scheme 1. 3,6‐Bis(5‐bromo‐2‐thienyl)‐2,5‐bis(2‐hexyldecyl)‐2,5‐dihydro‐pyrrolo[3,4‐*c*]pyrrole‐1,4‐dione (M1), 3,6‐bis(5‐bromo‐2‐thienyl)‐2,5‐bis(4‐hexyldodecyl)‐2,5‐dihydro‐pyrrolo[3,4‐*c*]pyrrole‐1,4‐dione (M2), and 2,5‐bis(trimethylstannyl) thiophene (M3) are all facilely synthesized monomers. The gel permeation chromatography traces of the two polymers against polystyrene standard are shown in Figure S1 in the Supporting Information. PDPPT3‐HDO and PDPPT3‐HDE had comparable number average molecular weight (*M*_n_) and polydispersity index (Đ) of 57 kDa, 1.5 and 62 kDa, 1.9, respectively. It is worth noting that the polymerization time of PDPPT3‐HDO was only 25 min to reach such molecular weight. The simple and mature synthesis procedures, large‐scale process of monomers, short polymerization time are all advantageous and important for potential mass production in industry. The short time polymerization to obtain high molecular weight CPs has been reported before.^28^ The reproducibility of the polymerization was investigated by repeating the reaction three times following the same conditions. As shown in Figure S2 and Table S2 in the Supporting Information, PDPPT3‐HDO polymers obtained from four batches exhibited similar molecular weight and OTFT device performance (see discussion below). PDPPT3‐HDO of higher molecular weights was also obtained by increasing the polymerization time, showing the controllability of the polymerization (Table S3, Supporting Information). The two polymers exhibited high thermal stability with decomposition temperatures (*T*_d_) above 350 °C according to thermogravimetric analysis (Figure S3a, Supporting Information) and no obvious thermal transitions were observed from differential scanning calorimetry measurements (Figure S3b, Supporting Information). The highest occupied molecular orbital and lowest unoccupied molecular orbital energy levels were −5.21 and −3.33 eV for PDPPT3‐HDO, −5.20 and −3.20 eV for PDPPT3‐HDE, respectively, as determined by film cyclic voltammetry (Figure S4 and Table S4, Supporting Information).

The structural design of increasing alkyl side chains is depicted in Figure 1b. In previous reports, alkylated dithienyl‐DPP units have been copolymerized with relatively large electron donors such as unsubstituted thienothiophene,^29^ bithiophene,^30^ dithienylethene,^31^ and diselenophenylethene^11^ to construct high mobility D‐A CPs, where alkyl side chains are less rich. These CPs were mostly processed from chlorinated solvents to fabricate OTFT devices. Herein instead of large size donors, we employed small size unsubstituted thiophene comonomer to afford higher density of alkyl side chains at the backbones, without introducing unnecessary structural complexity. Using this denser side chain strategy, both PDPPT3‐HDO and PDPPT3‐HDE are soluble in nonchlorinated solvent such as toluene, *o*‐xylene, and tetralin. It is noteworthy that though bearing the same alkyl side chain density, PDPPT3‐HDO and PDPPT3‐HDE displayed disparity in solubility (Table S5, Supporting Information). The solubility of the two polymers was measured by a standard calibration curve method using UV‐visabsorption,^32^ and the results are shown in Figure S5 and summarized in Table S5 in the Supporting Information. With a longer alkyl spacer from branching carbon to the backbone, PDPPT3‐HDO can be readily soluble in *o*‐xylene at room temperature to reach a solubility of 52.3 mg mL^−1^, while PDPPT3‐HDE can be dissolved in warm *o*‐xylene to reach a solubility of 8.7 mg mL^−1^. The spacer length‐dependent solubility has also been reported elsewhere.^33^, ^34^ Hence, by increasing the side chain density and tailoring the structure of the side chains, good solubility in nonchlorinated solvents can be ensured.

To probe whether *o*‐xylene is also a solvent for good wettability during film processing, the wetting property of PDPPT3‐HDO and PDPPT3‐HDE was investigated by comparing the contact angles of the polymer solutions in different solvents. As shown in Figure S6 in the Supporting Information, both PDPPT3‐HDO and PDPPT3‐HDE exhibited relatively small and comparable contact angles in *o*‐xylene and chlorobenzene as compared to those in tetralin and chloroform. *o*‐Xylene is thus a suitable nonchlorinated solvent for good wettability of the polymer solutions on the target Si/SiO_2_ substrates.

The UV‐vis–NIR (near‐infrared) absorption spectra of both polymers were measured in dilute *o*‐xylene solution and thin film (Figure S7 and Table S4, Supporting Information). Both polymers exhibited dual‐band absorptions which is a characteristicfeature of D‐A copolymers.^30^ Compared with PDPPT3‐HDE, PDPPT3‐HDO displayed a distinctly red‐shifted absorption maximum and absorption onset by approximately 15 nm in film, which indicates that the longer alkyl spacer between the branching point and backbone can reduce steric hindrance and leads to closer cofacial π–π stacking. Besides, temperature‐dependent UV‐vis–NIR absorption spectra of PDPPT3‐HDO and PDPPT3‐HDE were recorded (Figure S8, Supporting Information). Along with the increasing of temperature, both polymers show obvious blue‐shift in the absorption maximum. The temperature‐dependent absorption indicates that both polymers have pre‐aggregation at room temperature and reduced aggregation at high temperature in *o*‐xylene solution.

Film microstructures and morphologies of the two polymers were studied by 2D grazing incidence wide angle X‐ray scattering (2D‐GIWAXS), atomic force microscopy (AFM), and transmission electron microscopy (TEM). As shown in Figure S9 in the Supporting Information, drop cast PDPPT3‐HDO shows a relatively larger lamellar spacing of 21.53 Å than that of PDPPT3‐HDE (18.20 Å) because of its longer alkyl side chain. These lamellar distances are shorter than the length of fully extended alkyl side chains of PDPPT3‐HDE (≈31 Å) and PDPPT3‐HDO (≈36 Å) (Figure S10, Supporting Information), indicating interdigitation of alkyl side chains between two layers. The alkyl interdigitation extents of the two polymers are similar by comparing the difference between the lamellar distance of fully extended alkyls and the measured one by X‐ray diffraction (≈14 Å for PDPPT3‐HDO vs ≈12 Å for PDPPT3‐HDE). Compared with PDPPT3‐HDE, PDPPT3‐HDO has much shorter π–π stacking distance (3.61 Å vs 3.75 Å) because the branch point of the alkyl chains was moved away from the backbone and thus led to the more planar conjugated skeleton and closer cofacial π–π stacking, which is consistent with the result of absorption spectra above and is believed to enhance carrier mobilities.^11^, ^33^, ^34^, ^35^ Lamellar and π–π stacking coherence lengths (*L*_c_) of the two polymers were calculated by the Scherrer equation from the full‐width at half‐maximum of the (200) and (010) diffraction peaks.^36^ The larger lamellar and π–π stacking *L*_c_ of PDPPT3‐HDO (14.90 and 5.08 nm) than that of PDPPT3‐HDE (8.94 and 3.72 nm) suggests longer range of molecular order, which should yield more efficient charge transport. Besides, from tapping mode AFM topography images in Figure S11 in the Supporting Information, it is clear that the two polymers exhibit very different film morphologies. The spin cast film of PDPPT3‐HDE showed a segmented fibrillar structure of ≈20–40 nm diameter, while PDPPT3‐HDO film was composed of larger fiber‐like interconnected networks with bundles of ≈70–100 nm diameter. TEM results (Figure S12, Supporting Information) are in well accordance with the AFM data, revealing that film with higher crystallinity can be obtained from PDPPT3‐HDO instead of PDPPT3‐HDE.

We then fabricated OTFTs to evaluate the charge transport property of the polymers. PDPPT3‐HDE or PDPPT3‐HDO was used as semiconductor layer, *o‐*xylene as solvent, bar coating as film deposition method for top gate/bottom contact devices using unmodified Si/SiO_2_ as substrates. Detailed procedures of device fabrication are described in the Experimental Section. Bar coating is an attractive single‐step solution processing technique that can produce ordered thin films via meniscus effect.^14^, ^15^ Moreover, bar coating displays good compatibility with roll‐to‐roll (R2R) printing processes that is favorable for high throughput film production. The terms “parallel” and “vertical” are defined as when the coating direction of the wired‐bar is parallel or perpendicular to the source‐drain electric field. The transfer and output characteristics of bar‐coated OTFTs are shown in **Figure** 2 and Figure S13 in the Supporting Information. The detailed device performance data of the two polymers are summarized in **Table** 1. Both polymers exhibited typical p‐type transport behavior. PDPPT3‐HDO achieved a high saturation mobility of 9.24 cm^2^ V^−1^ s^−1^ and a linear mobility of 6.90 cm^2^ V^−1^ s^−1^ in parallel direction using the polymer by 25 min polymerization, among the highest mobilities reported for OTFTs processed from nonchlorinated solvents to date.^12^, ^21^, ^37^, ^38^ The performance of PDPPT3‐HDO from different batches of 25 min polymerization was investigated by testing the devices along the parallel direction. As shown in Figure S2 and Table S2 in the Supporting Information, PDPPT3‐HDO of all four batches displayed similar device parameters, suggesting acceptable batch‐to‐batch difference of the material. Molecular weight‐dependent device performance of PDPPT3‐HDO was also studied as shown in Figure S14 and Table S3 in the Supporting Information.

**Figure 2:**
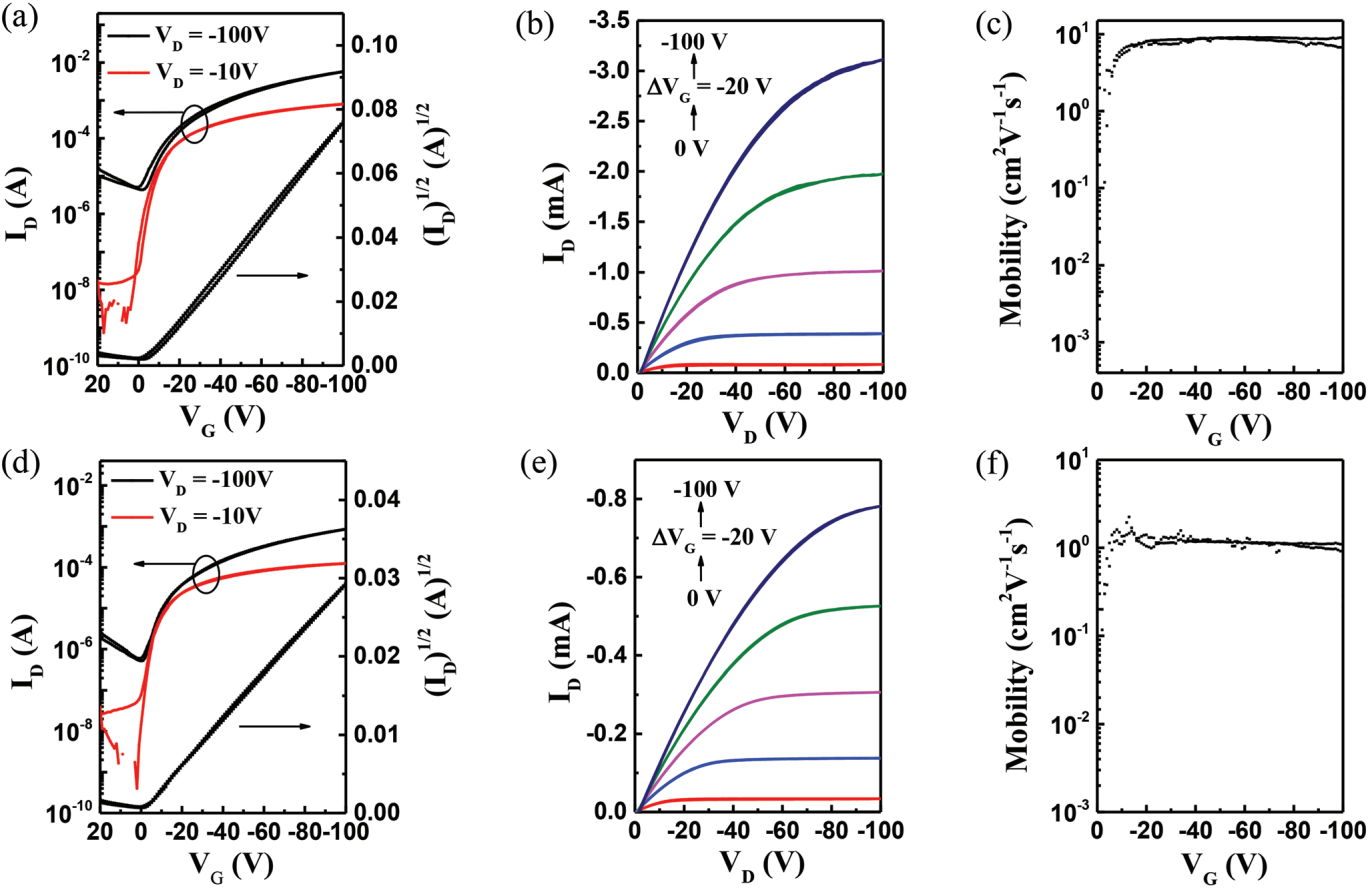
a,d) Typical transfer and b,e) output characteristics, and c,f) saturation mobility versus *V*_G_ of OTFT devices based on a–c) PDPPT3‐HDO and d–f) PDPPT3‐HDE by bar coating from *o*‐xylene solution in parallel direction.

**Table 1:** OTFT device performance data of bar‐coated PDPPT3‐HDO and PDPPT3‐HDE annealed at 120 °C

Polymer	Coating direction	*µ*_sat,avg_ (*µ*_sat,max_)^a)^[cm^2^ V^−1^ s^−1^]	*V*_T_ [V]^b)^	*I*_on_/*I*_off_ ^c)^	*µ*_lin,avg_ (*µ*_lin,max_)^d)^[cm^2^ V^−1^ s^−1^]	Dichroic ratio (*R*)^e)^(UV‐vis–NIR)	*r*_sat_ (*r*_lin_)^f)^
PDPPT3‐HDO	Parallel	8.17 ± 0.73 (9.24)	−8 to 1	10^3^–10^4^	5.66 ± 0.73 (6.90)	4.66	87% (86%)
	Vertical	2.73 ± 0.75 (3.86)	−12 to −2	10^2^–10^3^	1.89 ± 0.60 (3.00)		88% (75%)
PDPPT3‐HDE	Parallel	1.41 ± 0.14 (1.80)	−5 to 1	10^3^–10^4^	1.09 ± 0.16 (1.28)	1.01	79% (74%)
	Vertical	1.31 ± 0.25 (1.44)	−5 to 1	10^2^–10^3^	0.95 ± 0.22 (1.13)		83% (51%)

^a)^ Average and maximum (in brackets) saturation mobilities

^b)^ Threshold voltage

^c)^ Current on/off ratio

^d)^ Average and maximum (in brackets) linear mobilities

^e)^ Optical dichroic ratio *R* calculated from UV‐vis–NIR absorption

^f)^ Mobility reliability factor.

Notably, the extraction of mobility using the gradual channel approximation model with Equations (1) and (2) in the Supporting Information showed no “kink” or “double slope” phenomenon, and the highest saturation mobility has a reliability factor (*r*_sat_) of 87% (Table 1), indicating high quality transistors.^38^, ^39^, ^40^ The mobility of PDPPT3‐HDO obtained in vertical direction (*µ*_sat,max_ = 3.86 cm^2^ V^−1^ s^−1^, *µ*_lin,max_ = 3.00 cm^2^ V^−1^ s^−1^) is much lower, rendering highly anisotropic charge transport performance. Bar‐coated PDPPT3‐HDE, on the other hand, showed similar mobilities in parallel (1.80 cm^2^ V^−1^ s^−1^) and vertical (1.44 cm^2^ V^−1^ s^−1^) direction, without obvious anisotropy of charge transport.

The anisotropic electrical performance of bar‐coated PDPPT3‐HDO is related to its aligned film microstructure, as evidenced by GIWAXS and polarized optical absorption. According to the GIWAXS patterns, bar‐coated PDPPT3‐HDO has obvious lamella diffraction peaks in out‐of‐plane direction (Figure S15a, Supporting Information) with a d‐spacing of 21.27 Å, and (010) π–π stacking peak in in‐plane direction (**Figure** 3a) with a d‐spacing of 3.70 Å. The GIWAXS data suggest that the conjugated skeletons adopt favorable edge‐on orientation in thin film. Notably, the π–π diffraction peak can be recorded clearly when the incident light is parallel to the bar coating direction and disappears when the incident light is vertical to the bar coating direction, which suggests that the direction of π–π stacking is vertical to the bar coating direction. In other words, the polymer chains of PDPPT3‐HDO are uniaxially oriented in bar coating direction, yielding pronounced structural anisotropy and high charge carrier mobilities in the alignment direction. By using polarized UV‐vis–NIR spectroscopy, optical absorption anisotropies can be probed when the polarized incident beam is set to parallel or vertical to the coating direction, respectively (Figure 3b). The optical dichroic ratio *R* is a useful parameter to examine the anisotropy, defined by the ratio between the absorption of the 0–0 peak at around 844 nm in the parallel and vertical direction with respect to the coating direction of the wired‐bar (*R* = *A*_//_/*A*_⊥_).^41^ As shown in Figure 3b, PDPPT3‐HDO exhibited obvious anisotropic absorption with *R* = 4.66. The dichroism is the result of maximized absorption when molecules are aligned parallel to the incident beam (i.e., the coating direction), and quenched absorption when the long axis of molecules is vertical to the beam direction. An illustration of the packing mode of PDPPT3‐HDO is proposed based on the above GIWAXS and polarized optical absorption analysis (Figure 3c). As a comparison, PDPPT3‐HDE did not show obvious orientation in bar‐coated film as expected. There is no obvious anisotropy detected in GIWAXS when the X‐ray is set to parallel or vertical to the coating direction (Figures S15b and S16a, Supporting Information). The optical dichroic ratio *R* of bar‐coated PDPPT3‐HDE film equals 1.01 using polarized absorption (Figure S16b, Supporting Information). The microstructural and optical isotropy is in line with the charge transport isotropy of OTFT devices based on PDPPT3‐HDE (Table 1).

**Figure 3:**
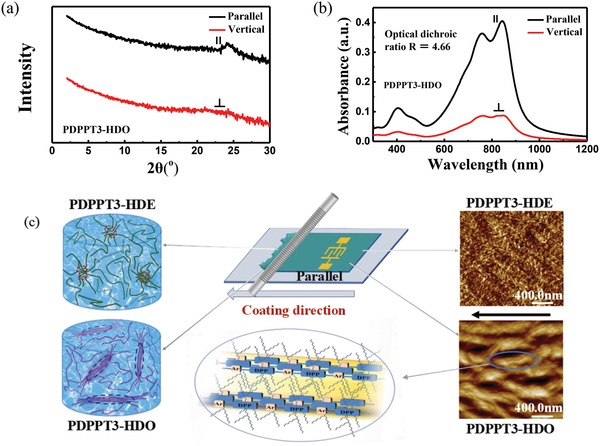
a) In‐plane GIWAXS patterns and b) polarized optical absorption of bar‐coated PDPPT3‐HDO film. c) The mimetic diagram of PDPPT3‐HDE (left top) and PDPPT3‐HDO (left bottom) aggregates in *o*‐xylene solution, sketch of the bar coating process (middle top), schematic illustration of proposed molecular packing of PDPPT3‐HDO in bar‐coated film (middle bottom), and AFM topography images (2 µm × 2 µm) of bar‐coated PDPPT3‐HDE (right top) and PDPPT3‐HDO (right bottom) from *o*‐xylene solution.

Aligned morphology of bar‐coated PDPPT3‐HDO film is also demonstrated by tapping mode AFM and TEM. As shown in Figure 3c, PDPPT3‐HDO was found to form aligned fibers extending along the coating direction, which is beneficial for charge transport along the alignment axial with reduced grain boundaries.^14^, ^42^ However, PDPPT3‐HDE formed meshlike morphology without obvious orientation. The bar‐coated PDPPT3‐HDO film was confirmed efficiently aligned by TEM (Figure S17a, Supporting Information) as well, and no significant enhancement in morphology order was observed for PDPPT3‐HDE (Figure S17b, Supporting Information) even shear force was applied during film preparation.

A plausible explanation of the significant charge transport anisotropy of bar‐coated PDPPT3‐HDO is related to the microstructure in aligned film. According to the proposed packing model of PDPPT3‐HDO (Figure 3c) as suggested by polarized absorption and GIWAXS results, the molecules are aggregated to fibrillar bundles with the polymer long axis being aligned parallel to the alignment direction of the fibers. Charge carrier transport along this direction is spatially of a intrachain manner enhanced by efficient conjugation, only infrequently assisted by interchain hopping. There are also much less grain boundaries in this parallel direction for carriers to trap in. On the contrary, transport perpendicular to the fiber alignment direction is largely interchain hopping‐controlled, resulting in the less efficient charge transport.

We assume that the different charge transport performances of PDPPT3‐HDE and PDPPT3‐HDO are due to their distinct morphology features inherited from their solution state aggregation structures (Figure 3c).^43^ The pre‐aggregates in solution were observed using TEM measurement on freeze‐dried samples (Figure S18, Supporting Information). PDPPT3‐HDO formed nanoscale fibrillar objects of longer aspect ratio (Figure S18a, Supporting Information), while PDPPT3‐HDE formed isotropic aggregates in *o*‐xylene solution (Figure S18b, Supporting Information). We proposed that these fibrils formed in solution are critical as potential nuclei for larger alignable fibers. The proposed different aggregates formed by PDPPT3‐HDE and PDPPT3‐HDO in solution are illustrated in Figure 3c. We suspect that the closer branching point of the alkyl chains to the backbone of PDPPT3‐HDE results in increased steric hindrance between conjugated skeleton to warp the intermolecular stacking, thus leads to nondirectional aggregates. Introducing alkyl chains with longer spacer could change the shape of the polymer aggregates in solution due to smaller steric hindrance and better solubility. The fact that PDPPT3‐HDO can be well dissolved in *o*‐xylene helps to extend the polymer chains in solution, and because of the strong intermolecular interaction and good planarity, the extended polymer chains tend to aggregate in a 1D stacking manner. Recently, Pei et al. have also reported that the solution state structures of CPs prior to film fabrication gave rise to optimized film microstructures and optoelectronic properties.^43^

For a high mobility over 9 cm^2^ V^−1^ s^−1^ of a CP, nonhopping‐limited mechanism is generally anticipated. This transport model is often characterized with low activation energy and even bandlike transport features.^44^, ^45^ To better understand the origin of the high mobility of PDPPT3‐HDO‐based parallel aligned devices, variable temperature measurement of OTFT mobility was carried out. Typical Arrhenius plots of the saturation mobility as a function of temperature are shown in **Figure** 4. The transport of PDPPT3‐HDO can be separated to two regimes, one temperature‐independent high mobility range (from 300 to 285 K) and the other linearly decreased mobility in the lower temperature range (from 275 to 240 K). In the operation window close to room temperature, the independence of mobility on temperature is an implication of bandlike, nonhopping transport mechanism. In a lower temperature range, the charge carriers are thermally activated with a relatively low activation energy (*E*_act_) of 50.4 meV. As a control, PDPPT3‐HDE‐based devices are thermally activated, hopping‐limited within the whole measurement range, with *E*_act_ of 96.3 meV.

**Figure 4:**
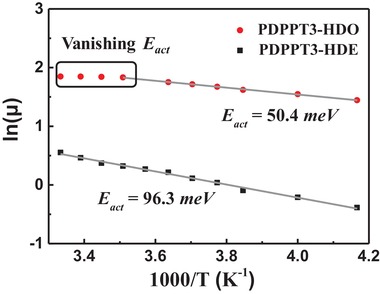
Arrhenius plot of mobility against temperature of bar‐coated PDPPT3‐HDO and PDPPT3‐HDE OTFT devices.

In conclusion, we have demonstrated that high mobility, simple structure, nonchlorinated solvent processable D‐A CPs can be obtained via denser alkyl side chains strategy. The good solubility in nonchlorinated solvent (≈50 mg mL^−1^ in *o*‐xylene) is feasible since minimized comonomer size results in increased density of alkyl side chains. The tailored length and density of the side chains of PDPPT3‐HDO are not only essential for increased solubility in nonchlorinated hydrocarbon solvents, but also determine self‐assembly properties of the polymer in solution and film as evidenced by optical and morphological investigation. Longer spacer between the branching carbon of side chain and the polymer backbone effectively regulates the aggregation behavior in solution, and the 1D pre‐aggregates further induce the formation of aligned fibers during bar coating. The aligned microstructure gives rise to anisotropic charge transport. Highest charge carrier mobility of 9.24 cm^2^ V^−1^ s^−1^ was reached when the electric field is parallel to the alignment direction, due to extended transport pathways with significantly reduced grain boundaries. We hope the denser alkyl side chain strategy for PDPPT3‐HDO can promote further studies on CPs for large‐scale production of high mobility OTFTs processed from nonchlorinated solvents.

## Conflict of Interest

The authors declare no conflict of interest.

## Supporting information

Supporting Information
