# Supplementary material for: A Simple Structure Conjugated Polymer for High Mobility Organic Thin Film Transistors Processed from Nonchlorinated Solvent
Source: Adv Sci (Weinh). 2019 Oct 29;6(24):1902412. doi: 10.1002/advs.201902412 (PMC6918096; doi:10.1002/advs.201902412)
Supplement: Supplementary file 1 — Supporting Information [file ADVS-6-1902412-s001.pdf]

Copyright WILEY-VCH Verlag GmbH & Co. KGaA, 69469 Weinheim, Germany, 2019.

ADVANCED SCIENCE
Open Access

Supporting Information

for *Adv. Sci.,* DOI: 10.1002/advs.201902412

A Simple Structure Conjugated Polymer for High Mobility Organic Thin Film Transistors Processed from Nonchlorinated Solvent

Zhongli Wang, Xianneng Song, Yu Jiang, Jidong Zhang, Xi Yu, Yunfeng Deng, Yang Han, Wenping Hu, and Yanhou Geng**

Copyright WILEY-VCH Verlag GmbH & Co. KGaA, 69469 Weinheim, Germany, 2019.

Supporting Information

A Simple Structure Conjugated Polymer for High Mobility Organic Thin Film Transistors Processed from Non-chlorinated Solvent

Zhongli Wang, Xianneng Song, Yu Jiang, Jidong Zhang, Xi Yu, Yunfeng Deng, Yang Han, Wenping Hu, Yanhou Geng**

Contents

1. General procedures and experimental details

2. Synthetic procedures and characterization

3. Fabrication and characterization of organic thin film transistors (OTFTs)

4. Complementary data

5. References

1. General procedures and experimental details

^1H NMR and ^{13}C NMR spectra were measured in chloroform-d ($CDCl_3$) at 25 °C with tetramethylsilane (TMS) as internal standard on a Bruker 400-MHz spectrometer. Elemental analysis was carried out on a FlashEA1112 elemental analyzer. Matrix-assisted laser desorption ionization time-of-flight (MALDI-TOF) mass spectra were characterized by a Bruker/AutoflexIII Smartbean MALDI mass spectrometer with 2-[(2E)-3-(4-tert-buthylphenyl)-2-methylprop-2-enylidene]malononitrile (DCTB) as the matrix in reflection mode. High temperature gel permeation chromatography (GPC) analysis of the polymers was conducted on a PL-GPC 220 system with 1,2,4-trichlorobenzene as the eluent and polystyrene as the standard at 150 °C. Thermogravimetric analysis (TGA) was conducted on a Perkin-Elmer TGA7 thermogravimetric analyzer at a heating rate of 10 °C min^{-1} at a nitrogen flow. Differential scanning calorimetry (DSC) was run on a Perkin-Elmer DSC7 at a heating/cooling rate of ±10 °C min^{-1} at a nitrogen flow. UV-vis-NIR absorption spectra were recorded on a Shimadzu UV3600 plus spectrometer. The optical bandgap was calculated according to film absorption onset (E_g^{opt} = $1240/\lambda_{onset}$ eV). Contact angle measurements were conducted by drop shape analysis (Kruss, Germany) with polymer solutions in different solvents as the test liquids on bare Si/SiO_2 substrates. Film cyclic voltammetry (CV) measurements were conducted in anhydrous acetonitrile with tetrabutylammonium hexafluorophosphate (Bu_4NPF_6, 0.1 mol L^{-1}) as the supporting electrolyte. The data were obtained on a CHI660 electrochemical analyzer with a three-electrode cell at a scan rate of 50 mV s^{-1}. A glassy carbon with 10 mm diameter, a Pt wire and a saturated calomel electrode (SCE) were used as working, counter and reference electrodes, respectively. To extract the energy levels of

polymer films, the potential was calibrated against ferrocene/ferrocenium (Fc/Fc^{+}) that was measured under the same conditions, which was 0.39 V versus SCE. The HOMO and LUMO energy levels were calculated according to the equations: E_{HOMO} = -(4.41 +E_{on}^{ox} $_{t}$) eV and E_{LUMO} = -(4.41 + E_{onset}^{re}) eV. Atomic force microscopy (AFM) measurements were carried out in tapping mode on a Bruker MultiMode 8 instrument. X-ray diffraction (XRD) of the thin films was measured with a Rigaku Smart Lab with Cu K_{α} source (λ = 1.54056 Å) in air. The two-dimensional grazing incidence wide angle X-ray scattering (2D GIWAXS) was measured at Shanghai Synchrotron Radiation Facility (SSRF) on beam line BL14B1 (λ = 0.124 nm) with a MarCCD area detector at incidence angle of 0.2^{o}. Transmission electron microscopy (TEM) images were recorded on a JEM-1011 transmission electron microscope with accelerating voltage of 100 KV and camera length of 160 cm. Variable temperature measurement of OTFT mobility was carried out by TTPX low temperature probe station (Lake Shore Cryotronics, Inc). The wired-bar was purchased from RK PrintCoat Instruments Ltd (KHC.11.1 K Bar), with a wet film thickness about 6 microns.

2. Synthetic procedures and characterization

All chemical reagents were purchased from Alfa-Aesar, Acros or Sigma-Aldrich and used as received unless otherwise noted. N,N-Dimethylformamide (DMF) was dried by CaH_2 before use. Toluene was distilled from sodium and benzophenone ketyl. Other solvents were used without further purification. M1, M3 and PDPPT3-HDE were prepared according to literature procedures. [1-3]

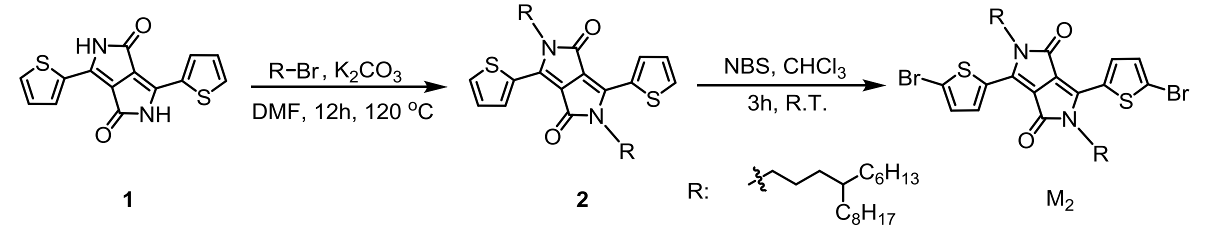

*Synthesis of 2,5-Dis(4-hexyldodecyl)-3,6-dithiophen-2-yl-pyrrolo[3,4-c]pyrrole-1,4-dione (**2**)*: A mixture of 3,6-dithienyl-2,5-diketopyrrolo[3,4-c]pyrrole (**1**) (0.30 g, 1.00 mmol), anhydrous potassium carbonate (K_2CO_3) (0.41 g, 3.00 mmol), DMF (10 mL), 7-(3-bromopropyl) pentadecane (1.00 g, 3.00 mmol) were added to a three-neck roundbottom flask. The mixture was stirred for about 12 h at 120 °C and then cooled to room temperature. The DMF was removed under vacuum, then the residue was dissolved in dichloromethane, washed with brine and dried with magnesium sulfate ($MgSO_4$). After the solvent was removed, the organic phase was purified by chromatography (silica gel, dichloromethane: petroleum ether = 1:1) to give a dark red solid (0.50 g, yield: 67%). ^1H NMR (400 MHz, $CDCl_3$, δ): 8.94 (dd, J_1 = 4.0 Hz, J_2 = 1.2 Hz, 2H), 7.63 (dd, J_1 = 5.2 Hz, J_2 = 1.2 Hz, 2H), 7.30-7.28 (m, 2H), 4.04 (t, J = 8.0 Hz, 4H), 1.75-1.67 (m, 4H), 1.39-1.22 (m, 54H), 0.89-0.85 (m, 12H); ^{13}C NMR (100 MHz, $CDCl_3$, δ): 161.28, 139.96, 135.30, 130.54, 129.81, 128.59, 107.69, 42.54, 37.15, 33.56, 31.94, 30.48, 30.10, 29.75, 29.68, 29.38, 27.05, 26.70, 26.65, 22.70, 14.13. Elemental Anal. calcd. for $C_{50}H_{80}N_2O_2S_2$: C 74.57, H 10.01, N 3.48, S 7.96; Found: C 74.71, H 10.04, N 3.38, S 8.60. MS (MALDI-TOF) *m/z*: calcd. for $C_{50}H_{80}N_2O_2S_2$: 804.57; Found. 804.72.

Synthesis of 3,6-Bis(5-bromo-2-thienyl)-2,5-bis(4-hexyldodecyl)-2,5-dihydro-pyrrolo[3,4-c] pyrrole-1,4-dione (M2): A solution of 2 (0.54 g, 0.67 mmol) in chloroform (21 mL) were added to a Schlenk flask, and N-bromosuccinimide (NBS) (0.30 g, 3.00 mmol) was added slowly at 0 °C Then the mixture was

gradually raised to room temperature and stirred for about 3 h in the absence of light. After the solvent was removed, the organic phase was purified by chromatography (silica gel, dichloromethane: petroleum ether = 1:2) to give a dark purple solid (0.40 g, yield: 62%).^{1}H NMR (400 MHz, $CDCl_3$, δ): 8.69 (d, *J* = 4.4 Hz, 2H), 7.24 (d, *J* = 4.0 Hz, 2H), 3.95 (t, *J* = 8.0 Hz, 4H), 1.71-1.64 (m, 4H), 1.38-1.23 (m, 54H), 0.89-0.85 (m, 12H); ^{13}C NMR (100 MHz, $CDCl_3$, δ): 160.94, 138.94, 135.40, 131.63, 131.14, 119.13, 107.78, 42.60, 37.10, 33.53, 31.94, 30.40, 30.11, 29.76, 29.68, 29.38, 27.03, 26.72, 26.66, 22.71, 22.70, 14.14. Elemental Anal. calcd. for $C_{50}H_{78}Br_2N_2O_2S_2$: C 62.36, H 8.16, N 2.91, S 6.66; Found: C 62.26, H 8.26, N 2.81, S 7.31. MS (MALDI-TOF) *m/z*: calcd. for $C_{50}H_{80}N_2O_2S_2$: 962.39; Found. 962,61.

Synthesis of PDPPT3-HDO: M2 (160.0 mg, 0.17 mmol), M3 (69.4 mg, 0.17 mmol), tris(dibenzylideneacetene)dipalladium(0) (4.6 mg, 3.00 mol%), triphenylphosphine (5.2 mg, 12.00 mol%), anhydrous toluene (3.0 mL) and anhydrous DMF (0.3 mL) were added to a Schlenk tube under argon. The mixture was stirred for 25 min at 115 °Ɑn the absence of light. 2-Bromobenzene (0.5 mL) was added as end-capper, and the reaction was stirred for another 12 h. After cooling to room temperature, the mixture was added dropwise to 200 mL methanol. The solid was collected and Soxhlet-extracted with ethanol, acetone, and hexane. PDPPT3-HDO was obtained as a black solid after vacuum drying (142.0 mg, yield 96%). Elemental Anal. calcd. for $(C_{56}H_{86}N_2O_2S_3)_n$: C 73.47, H 9.47, N 3.06, S 10.51; Found: C 72.74, H 9.10, N 2.95, S 11.32.

3. Fabrication and characterization of organic thin film transistors (OTFTs)

Top-gate/bottom-contact (TG/BC) OTFT devices were fabricated on Si/SiO_2 wafers. The substrates were cleaned by an ultrasonic cleaner with deionized water, acetone and isopropanol, then dried under a nitrogen flow. The Si/SiO_2 substrates were used without modification. Au (30 nm) was vacuum-evaporated on the silicon substrates as source and drain electrodes with an interdigitated electrode pattern (W/L = 70, W = 5.6 mm, L = 80 μm), and functionalized by pentafluorothiophenol (PFBT) to reduce injection barrier of holes. The semiconductor layer was deposited by bar coating from *o*-xylene solution at a concentration of 4.0 mg mL^{-1} at a speed of 40 mm s^{-1} in nitrogen and substrate temperature of 80 °C Films were then thermally annealed for 10 min at 120 °C in nitrogen. Afterwards, CYTOP (Asahi Glass, type CTL-809M) dielectric layer with a thickness of about 900 nm was spin coated atop at 2000 rpm for 2 min, and annealed at 100 °C for 40 min. Finally, Al (80 nm) was vacuum-evaporated as gate electrodes. The devices were measured in ambient conditions with Keysight B1500A analyzer. The devices were measured in ambient conditions with Keysight B1500A analyzer. Saturation and linear mobilities were calculated according to equation (1) and (2), respectively:

$$\mu_{sat}(V_G) = \frac{\partial I_{D,sat}}{\partial V_G} \cdot \frac{L}{WC_i} \cdot \frac{1}{(V_G - V_T)} \qquad (1)$$

and

$$\mu_{lin} = \frac{\partial I_D}{\partial V_G} \cdot \frac{L}{WC_iV_D} \qquad (2)$$

where W/L is the channel width/length, I_D the drain-source current, μ the field-effect mobility, C_i the capacitance per unit area of the gate dielectric layer, V_G, V_D and V_T the gate voltage, drain voltage

and threshold voltage, respectively. The mobility reliability factor was calculated using equations in the literature.[4]

4. Complementary data

Table S1. Summary of the mobility of organic thin film transistors processed from non-chlorinated solvents in recent ten years.

Year	Materials	$\mu_{h,max}$ [$cm^2 V^{-1} s^{-1}$]	Solvents	Design strategies	Device structure a)	Ref
2009	PTHOT	0.007	Tetrahydrofuran	Side chain engineering	BG/TC	5
2010	PBTDT-12	0.32	Hexane	Other structural design	BG/BC	6
2013	P3TEGT	0.000035	Water	Side chain engineering	BG/BC	7
2013	PTDPPTFT4	2.1	*p*-Xylene	Side chain engineering	BG/TC	8
2014	PTDPPTFT4	3.94	*p*-Xylene (25%)/ tetrahydronaphthalene	Screening of non-chlorinated solvent	BG/TC	9
2014	P-DPP-BTT(1) -SVS(9)	6.51	Xylene	Irregular synthetic approaches	BG/TC	10
2014	pDTTG-DTBT (C16)	0.26	1,3,5-Trimethylbenzene:1-methylnaphthalene (1:1)	Side chain engineering	TG/BC	11
2015	PFBT-Th4(1,4)	1.06	d-Limonene	Screening of non-chlorinated solvent	BG/TC	12
2015	PDPP-TT	2.7	Butyl acetate	Sub-microparticles formation	BG/BC	13
2015	PDPP-SVS	2.5	Water, ethanol	Smart Surfactant Engineering	BG/BC	14
2015	P-DPP-TT(7)-SVS(3)	1.03	Butanol	Sub-microparticles formation	BG/BC	15
2015	PDPP–TVS–C29	8.2	Tetralin	Irregular synthetic approaches	BG/TC	16
2016	PBTTT	0.19	Water	Smart Surfactant Engineering	BG/TC	17
2016	PDPPMT-2T	12.5	Toluene/diphenyl ether (2%)	Irregular synthetic	BG/BC	18

				approaches		
2016	PDPP2TPCL	0.81	Toluene/diphenyl ether (2%)	Other structural design	BG/BC	19
2017	LGC-D118	2.60	2-Methyltetrahydrofuran	Side chain engineering	TG/BC	20
2017	PFDPPTT-Si	1.87	Toluene	Control solubilizing comonomer	BG/TC	21

Table S1. (Contd.)

Year	Materials	$\mu_{h,max}$ [cm^2 V^{-1} s^{-1}]	Solvents	Design strategies	Device structure [a)]	Ref
2017	PIDSeBT	6.4	Tetralin	Side chain engineering	TG/BC	22
2017	PDPP2DT-F2T2	1.28	1,2,4-Trimethylbenzene	Backbone configuration	TG/BC	23
2017	PPDT2FBT-A	0.01	Ethanol	Side chain engineering	BG/TC	24
2018	Lin-CZ-T	0.15	Tetrahydrofuran/hexane (15%)	Backbone configuration	BG/TC	25
2018	IDT-BT	0.785	Mesitylene/acetophenone (5%)	Screening of non-chlorinated solvent	TG/BC	26
2018	PTTFDPP-TT	0.12	Toluene	Irregular synthetic approaches	BG/BC	27
2018	DPP-10C$_5$DE	2.76	Tetralin	Control solubilizing comonomer	BG/TC	28
2019	C$_8$-BTBT	5.4	Mesitylene	Screening of non-chlorinated solvent	TG/BC	29

[a)] BG = bottom gate, BC = bottom contact, TG = top gate, TC = top contact

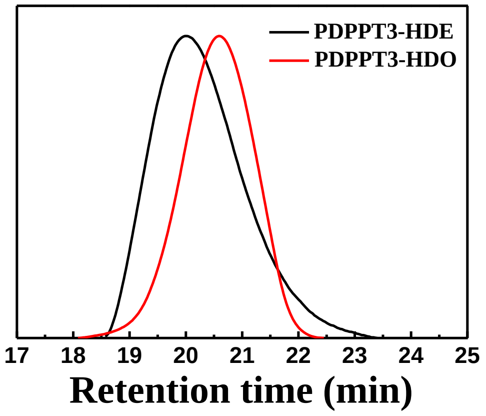

Figure S1. GPC curves of the polymers.

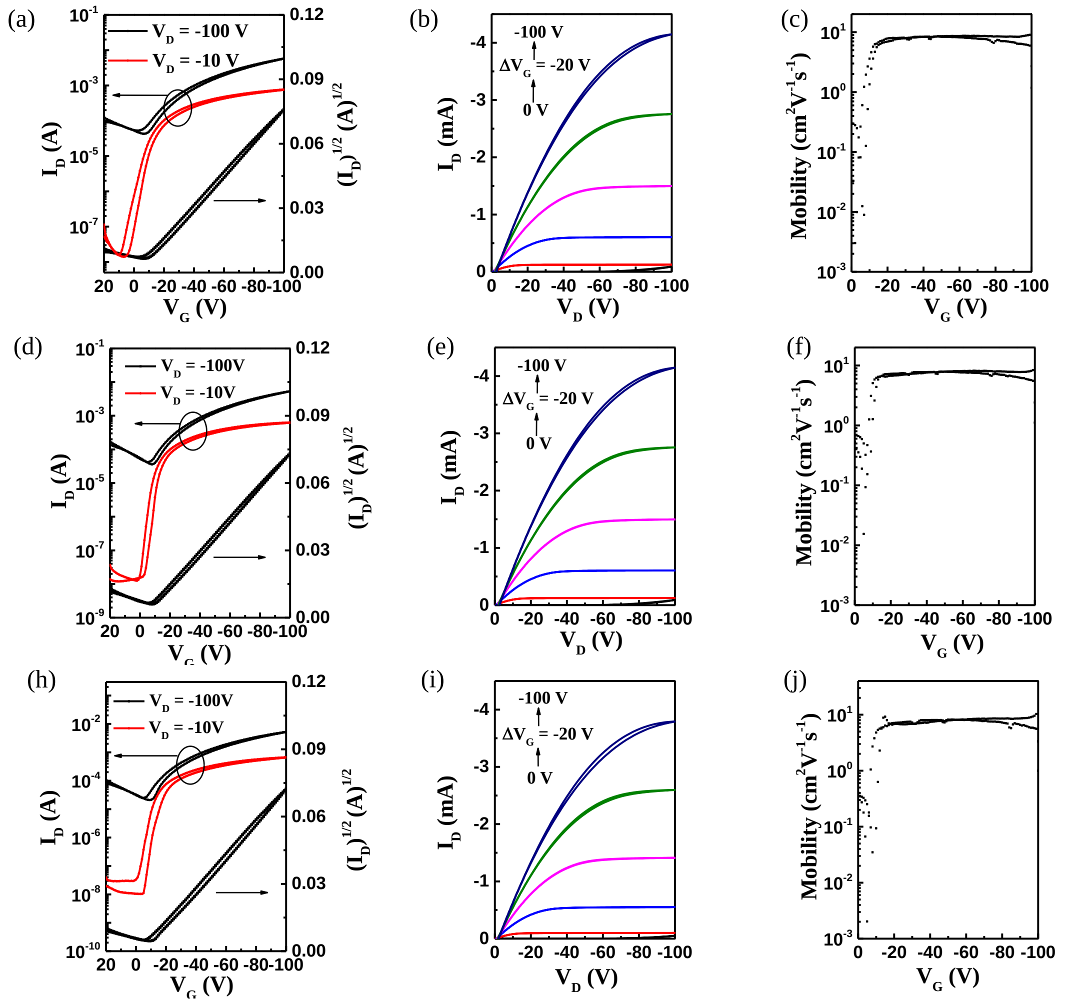

Figure S2. Typical transfer (a, d, h), output (b, e, i) curves and mobility versus V_{GS} (c, f, j) plots of PDPPT3-HDO OTFT devices based on entry 2 (a-c), entry 3 (d-f) and entry 4 (h-j) as mentioned in Table S2 by bar-coating in parallel direction from *o*-xylene.

Table S2. OTFT device performance data and molecular weights of PDPPT3-HDO from different batches by 25 min polymerization.

Batch entry	M_w [kDa]*	$\mu_{sat,avg}$ ($\mu_{sat,max}$) [$cm^2 V^{-1} s^{-1}$]	V_T [V]	I_{on}/I_{off}	$\mu_{lin,avg}$ ($\mu_{lin,max}$) [$cm^2 V^{-1} s^{-1}$]	Coating direction
1	84	8.17±0.73 (9.24)	-8 to 1	10^3-10^4	5.66±0.73 (6.90)	Parallel

2	91	7.35±0.95 (8.69)	-5 to 8	10^2-10^3	5.61±0.40 (6.24)	Parallel
3	83	7.38±0.57(8.19)	-9 to -4	10^2-10^3	5.77±0.44 (6.05)	Parallel
4	88	7.66±0.75 (8.67)	-1 to 8	10^2-10^3	5.33±0.49 (5.98)	Parallel

*Weight average molecular weight (M_w). The high molecular weight fraction is the key prerequisite for achieving such high mobility by bridging the crystalline domains.[30,31] M_w is a more suitable expression of the reproducibility to obtain the important high molecular weight fraction.

Table S3. OTFT device performance data and molecular weights of PDPPT3-HDO by different polymerization times.

Polymerization time [min]	M_n [kDa]/$Đ$	$\mu_{sat,avg}$ ($\mu_{sat,max}$) [$cm^2 V^{-1} s^{-1}$]	V_T [V]	I_{on}/I_{off}	$\mu_{lin,avg}$ ($\mu_{lin,max}$) [$cm^2 V^{-1} s^{-1}$]	Coating direction
25	57/1.5	8.17±0.73 (9.24)	-8 to 1	10^3-10^4	5.66±0.73 (6.90)	Parallel
40	78/3.5	2.78±0.09 (2.90)	-8 to -1	10^2-10^3	2.49±0.16 (2.62)	Parallel
60	84/3.5	3.71±0.17(3.84)	-12 to -7	10^3-10^4	2.57±0.02 (2.60)	Parallel
70	101/3.6	3.13±0.50 (3.63)	-7 to -6	10^2-10^3	2.51±0.59 (3.09)	Parallel

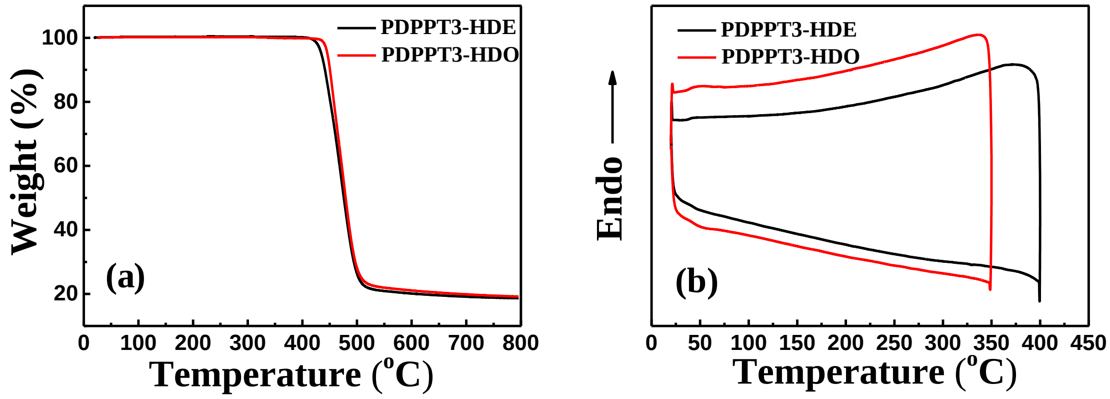

Figure S3. TGA (a) and DSC (b) curves of the polymers.

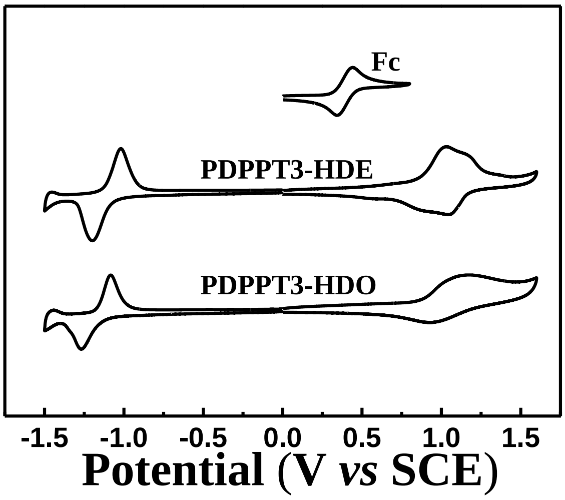

Figure S4. Film cyclic voltammograms (CV) of the polymers.

Table S4. T_d, optical and electrochemical properties of the polymers.

Polymer	λ_{max} [nm]		Bandgap [eV]		LUMO [eV][b)]	HOMO [eV][b)]	T_d [°C]
	Solution	Film	Optical[a)]	CV			
**PDPPT3-HDO**	843, 767, 404	853, 768, 407	1.33	2.00	-3.20	-5.20	355
**PDPPT3-HDE**	835, 788, 419	839, 794, 422	1.34	1.88	-3.33	-5.21	436

[a)]The optical bandgaps calculated from the film absorption onsets.

[b)]The HOMO and the LUMO energy levels were calculated according to E_{HOMO} = -(4.41 + E_{onset}^{ox}) eV and E_{LUMO} = -(4.41 + E_{onset}^{re}) eV, in which E_{onset}^{ox} and E_{onset}^{re} represent oxidation and reduction onset potentials of the polymers versus SCE, respectivily.

Table S5. Solubility of the polymers in *o*-xylene.

**Polymer**	**PDPPT3-HDO**	**PDPPT3-HDE**
Solubility in *o*-xylene	52.3 mg mL^{-1}	8.7 mg mL^{-1}

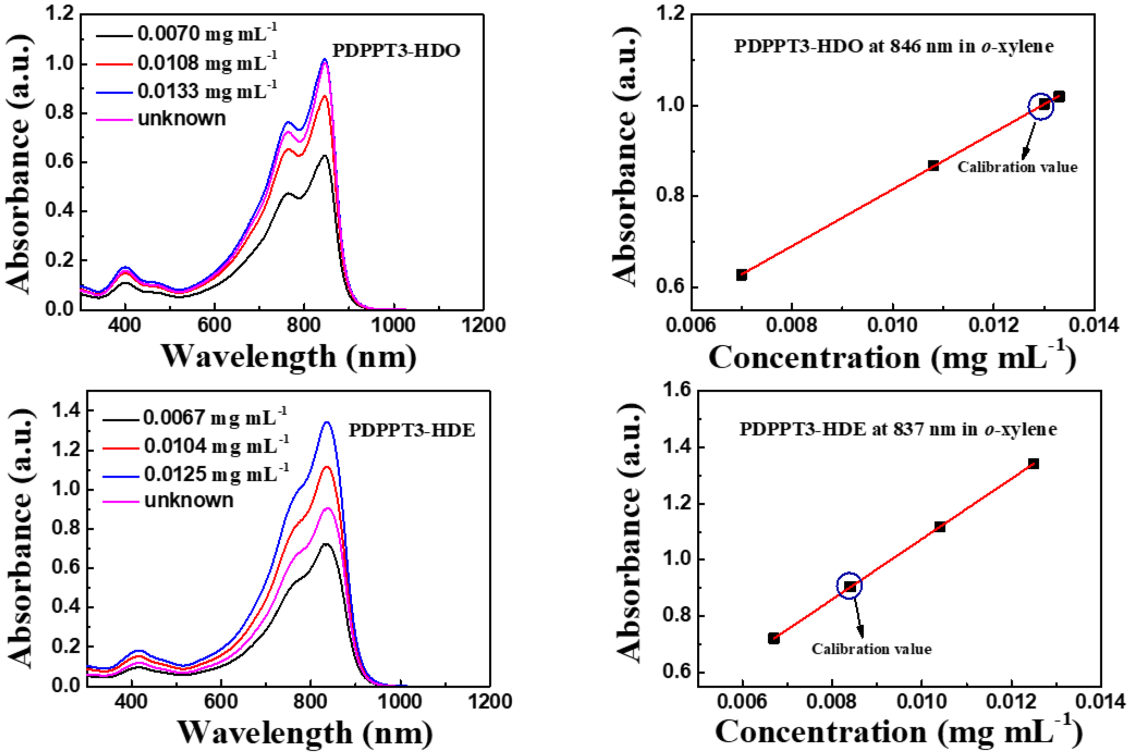

Figure S5. The solubility of two polymers in *o*-xylene can be inferred by absorption spectrum: (Left) UV-vis-NIR absorption spectra of two polymers at a determined concentration in dilute *o*-xylene solution; (Right) the functional relationship between the absorption and concentration of polymers at a certain wavelength. The unknown PDPPT3-HDO based sample was prepared by dilution of 4000 times from saturated solution. The unknown PDPPT3-HDE based sample was prepared by dilution of 1000 times from saturated solution.

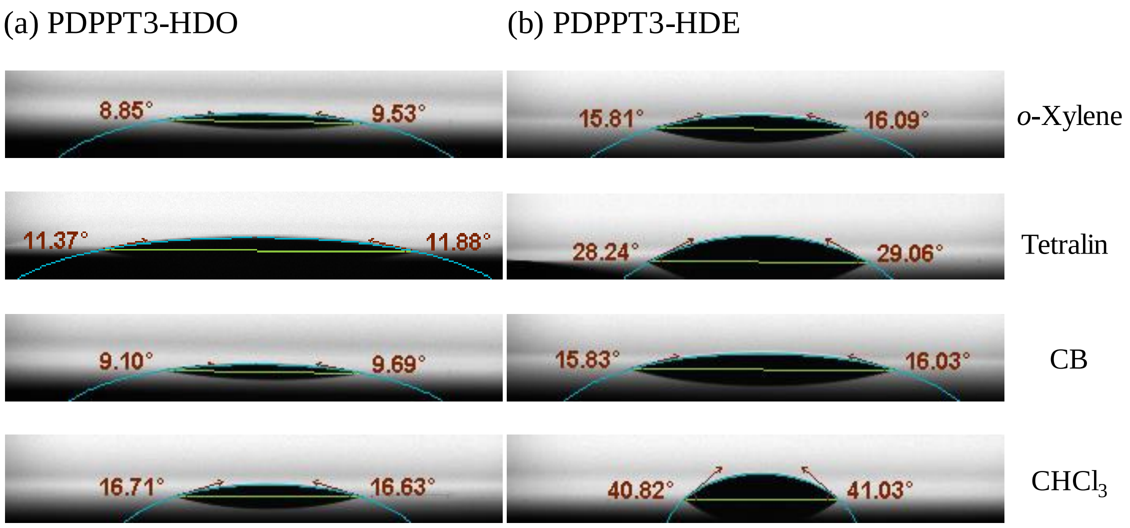

Figure S6. Contact angle of different polymer solutions on Si/SiO_2 substrate.

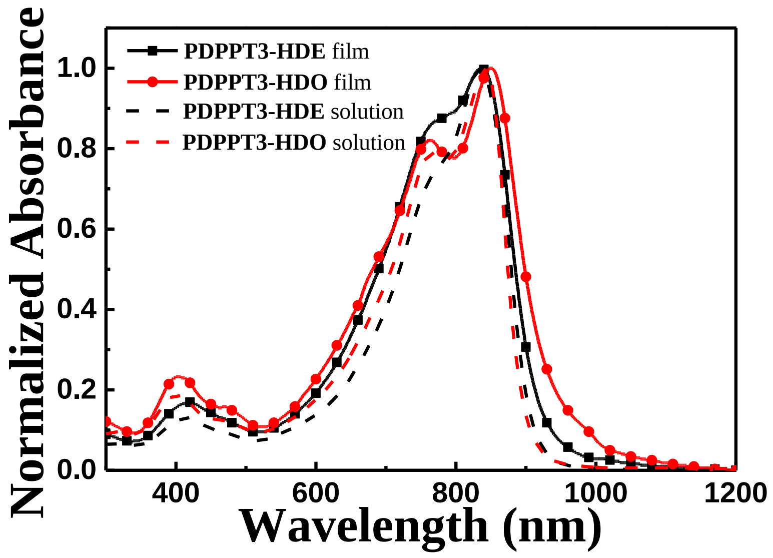

Figure S7. UV-vis-NIR absorption spectra of the polymers in solution and film.

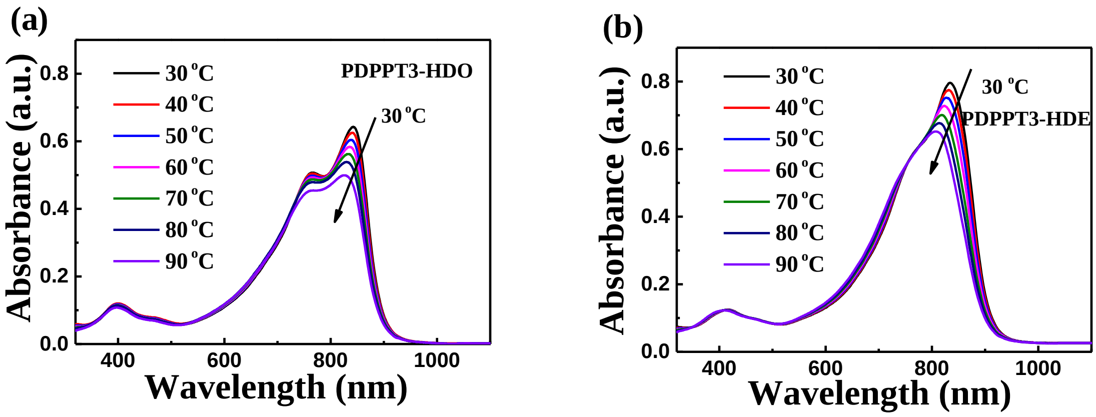

Figure S8. Temperature-dependent UV-vis-NIR absorption spectra of PDPPT3-HDO (a) and PDPPT3-HDE (b) in dilute *o*-xylene solution.

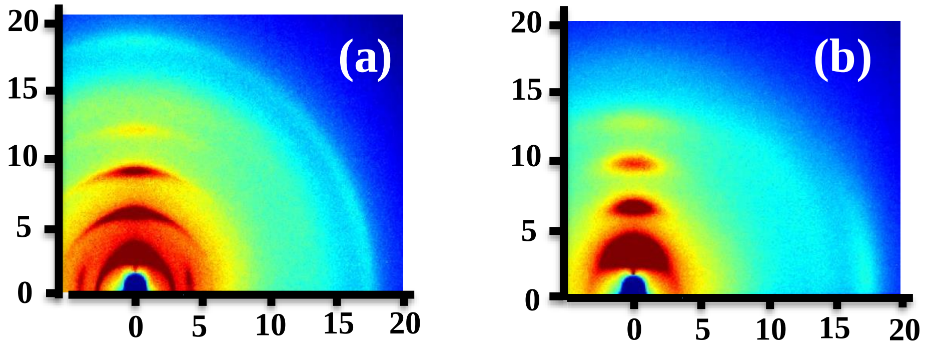

	PDPPT3-HDO	PDPPT3-HDE
Lamella spacing (Å)	21.53	18.20
(200) Peak fwhm (1/nm)	0.422	0.703
$L_{c,\ Lamella}$ (nm)	14.9	8.94
π-π stacking distance (Å)	3.61	3.75
(010) Peak fwhm (1/nm)	1.24	1.69

$L_{c,\ \pi\text{-}\pi}$ (nm)	5.08	3.72

Figure S9. 2D-GIWAXS patterns of drop cast PDPPT3-HDO (a) and PDPPT3-HDE (b) film, and corresponding data in a summary table.

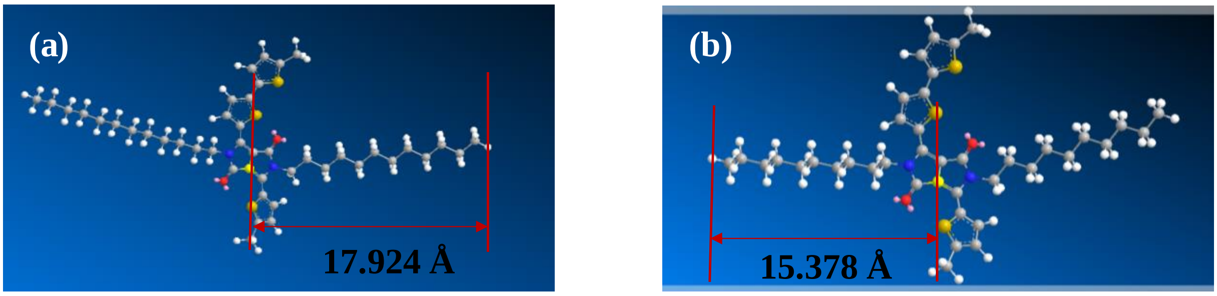

Figure S10. Lamellar distance of PDPPT3-HDO (a) and PDPPT3-HDE (b) for fully extended alkyls according to DFT calculation.

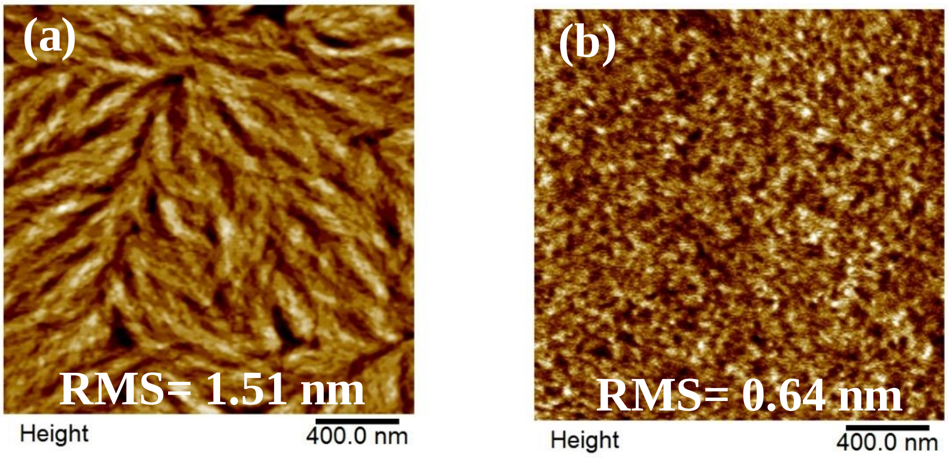

Figure S11. AFM topography images (2 μm × 2 μm) of PDPPT3-HDO (a) and PDPPT3-HDE (b) film by spin-coating from *o*-xylene solution.

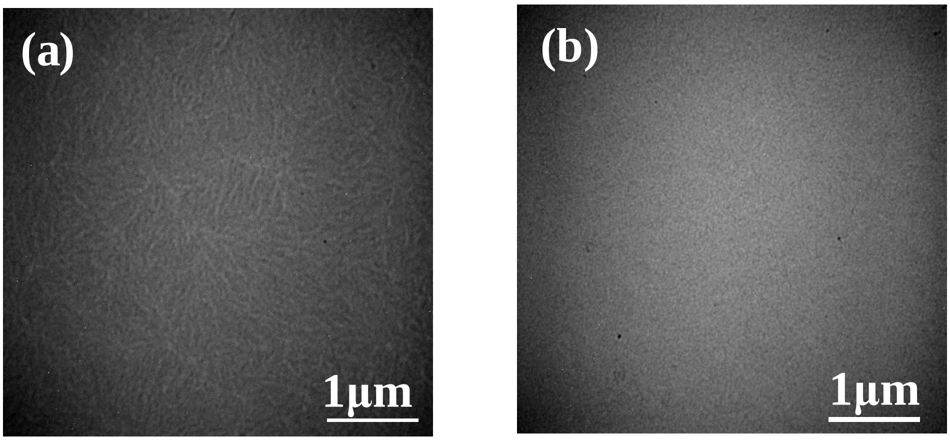

Figure S12. TEM images of PDPPT3-HDO (a) and PDPPT3-HDE (b) film by spin-coating from *o*-xylene solution.

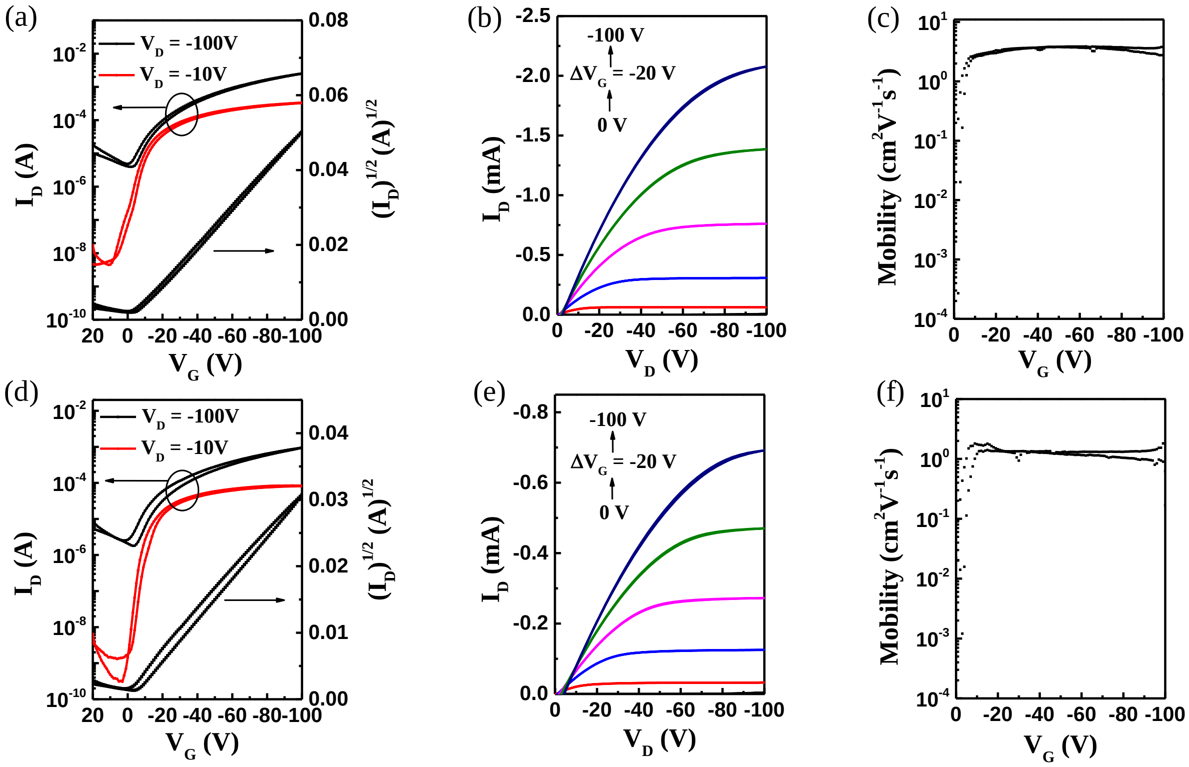

Figure S13. Typical transfer (a, d) and output (b, e) characteristics, and saturation mobility versus V_G (c, f) of OTFT devices based on PDPPT3-HDO (a-c) and PDPPT3-HDE (d-f) by bar coating from *o*-xylene solution in vertical direction.

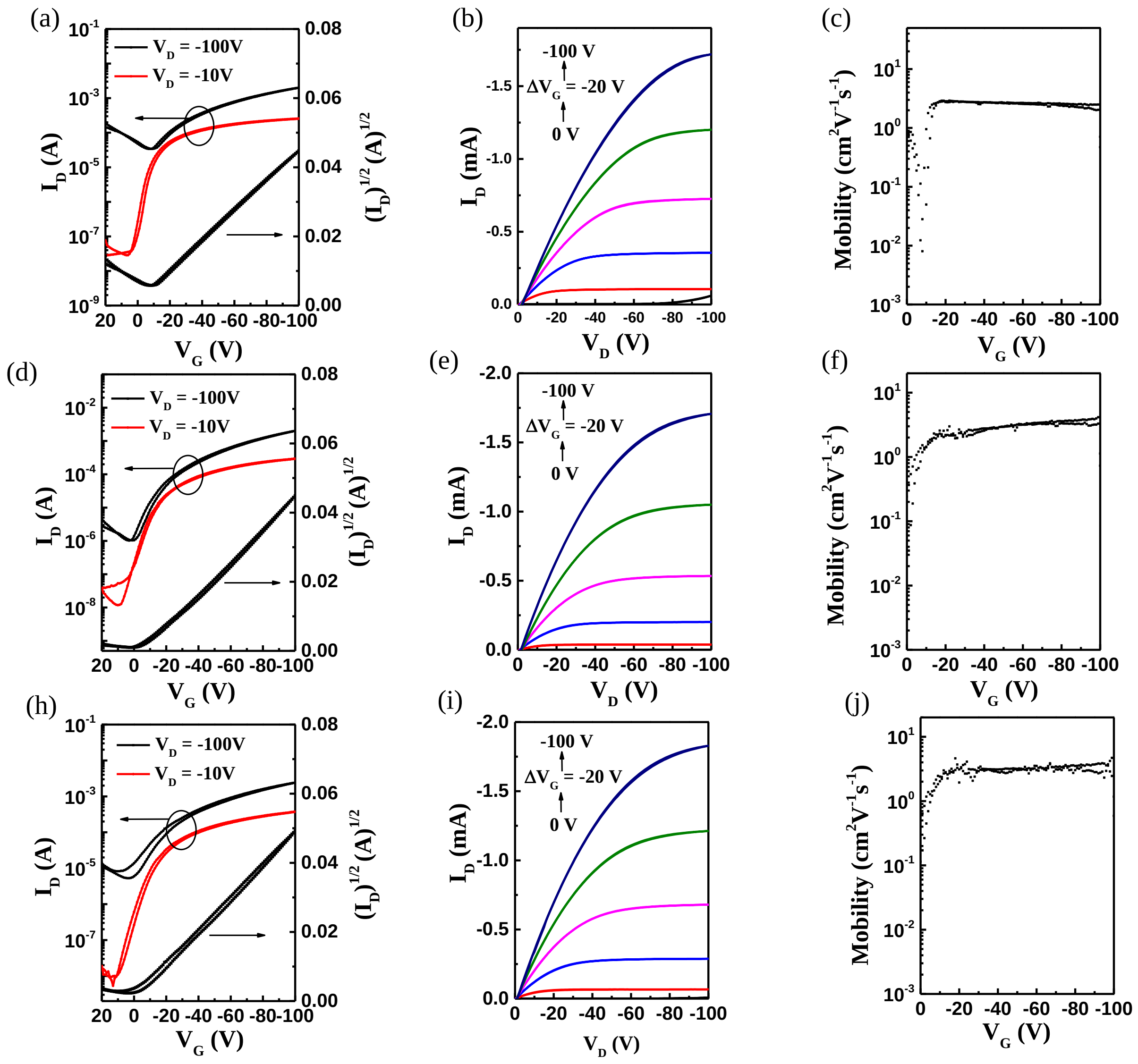

Figure S14. Typical transfer (a, d, h), output (b, e, i) curves and mobility versus V_{GS} (c, f, j) of PDPPT3-HDO OTFT devices based on polymers from 40 min (a-c), 60 min (d-f) and 70 min (h-j) by bar-coating in parallel direction from *o*-xylene.

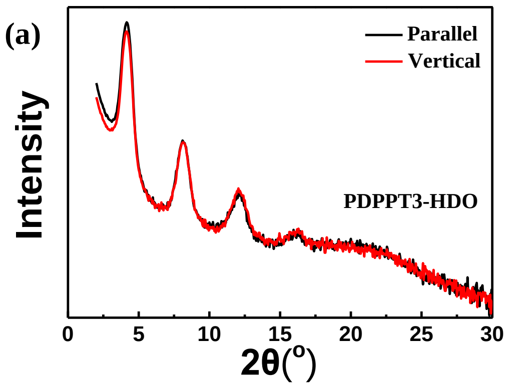

Figure S15. Out-of-plane GIWAXS patterns of bar-coated PDPPT3-HDO (a) and PDPPT3-HDE (b) film.

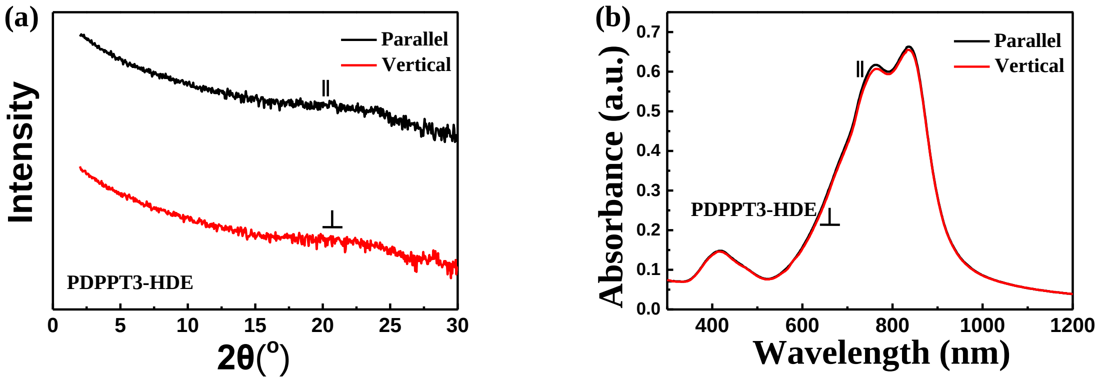

Figure S16. In-plane GIWAXS patterns (a) and polarized optical absorption (b) of bar-coated PDPPT3-HDE.

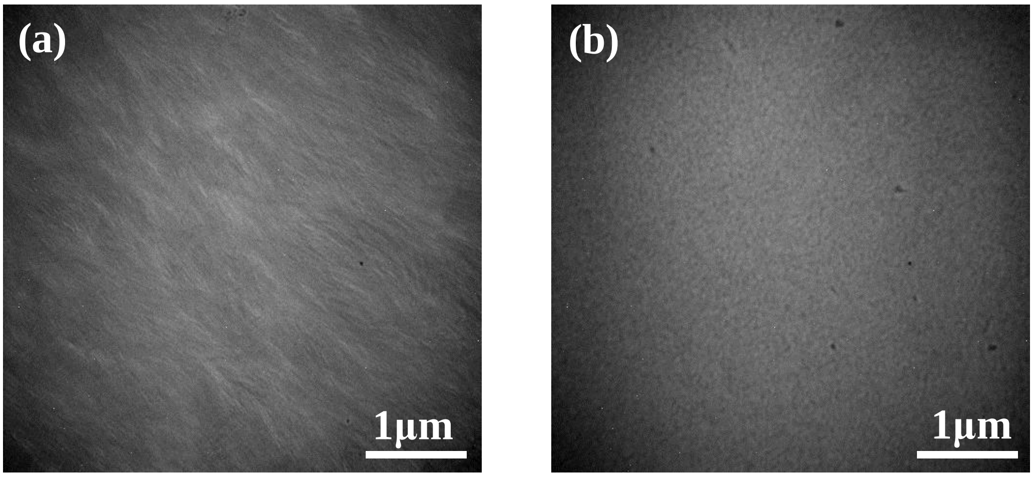

Figure S17. TEM images of bar-coated PDPPT3-HDO (a) and PDPPT3-HDE (b) film from *o*-xylene solution.

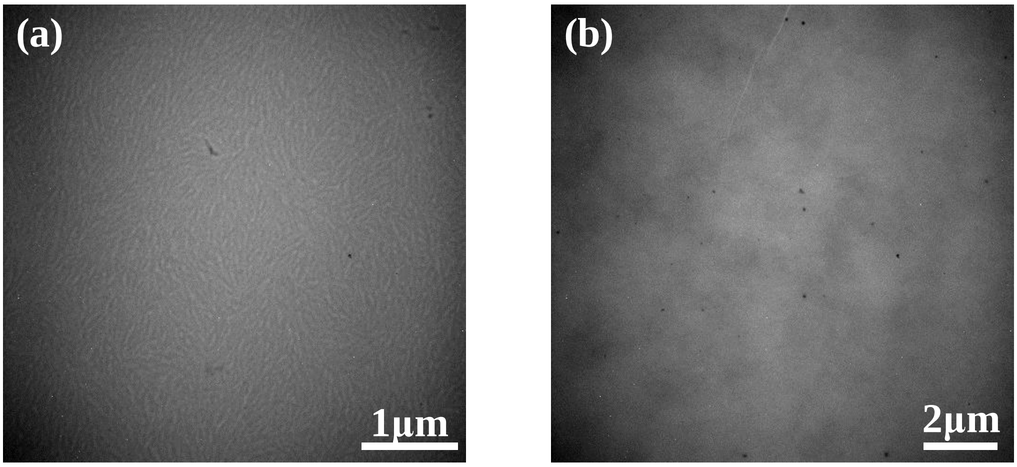

Figure S18. TEM images of freeze-dried PDPPT3-HDO (a) and PDPPT3-HDE (b) from 4 mg mL^{-1} *o*-xylene solution.

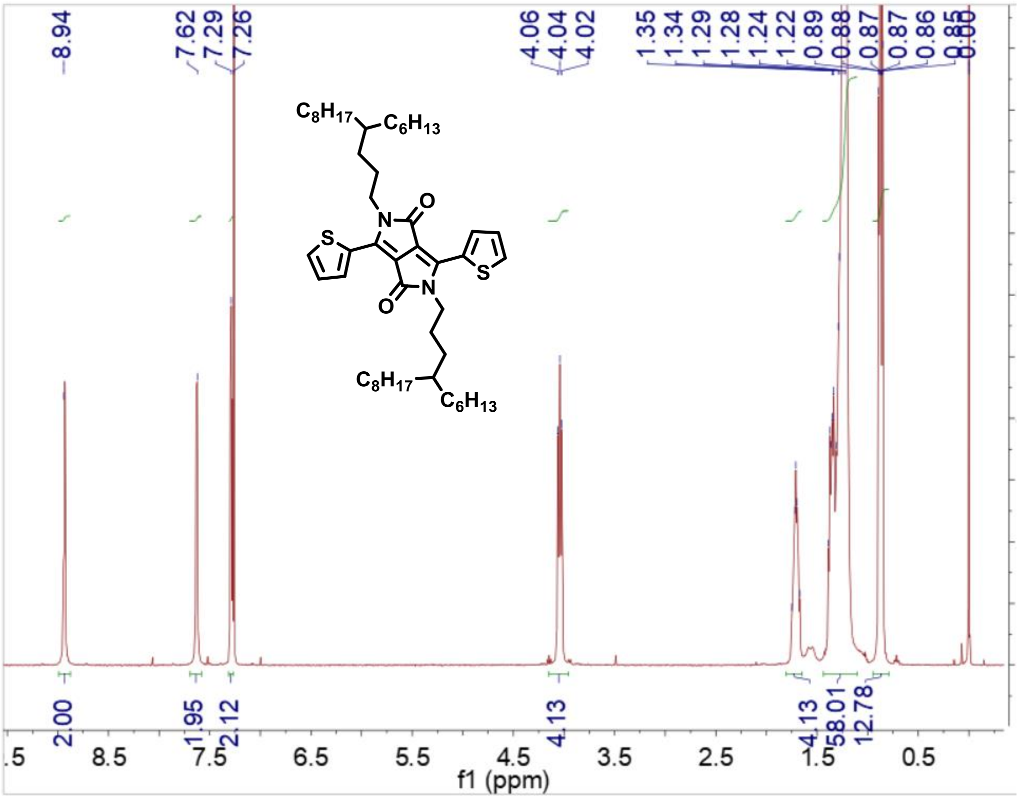

Figure S19. ^{1}H NMR spectrum (400 MHz, $CDCl_3$, 25 °C) of **2**.

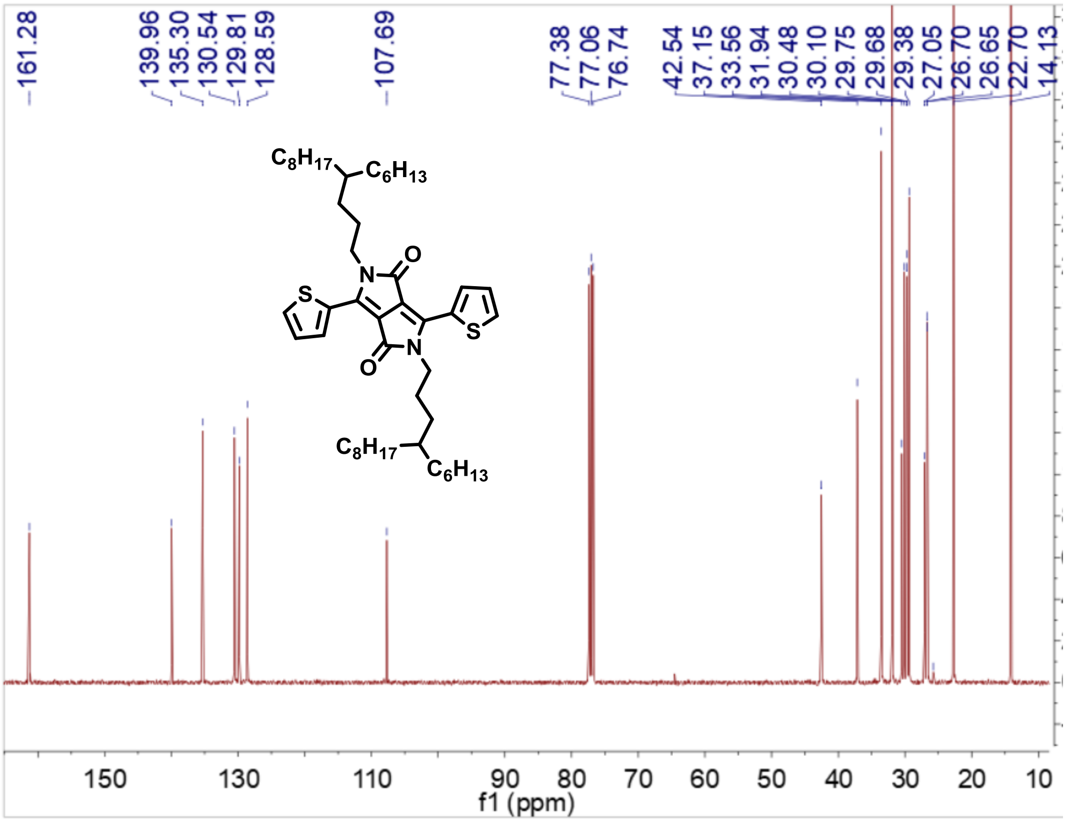

Figure S20. ^{13}C NMR spectrum (100 MHz, $CDCl_3$, 25 °C) of **2**.

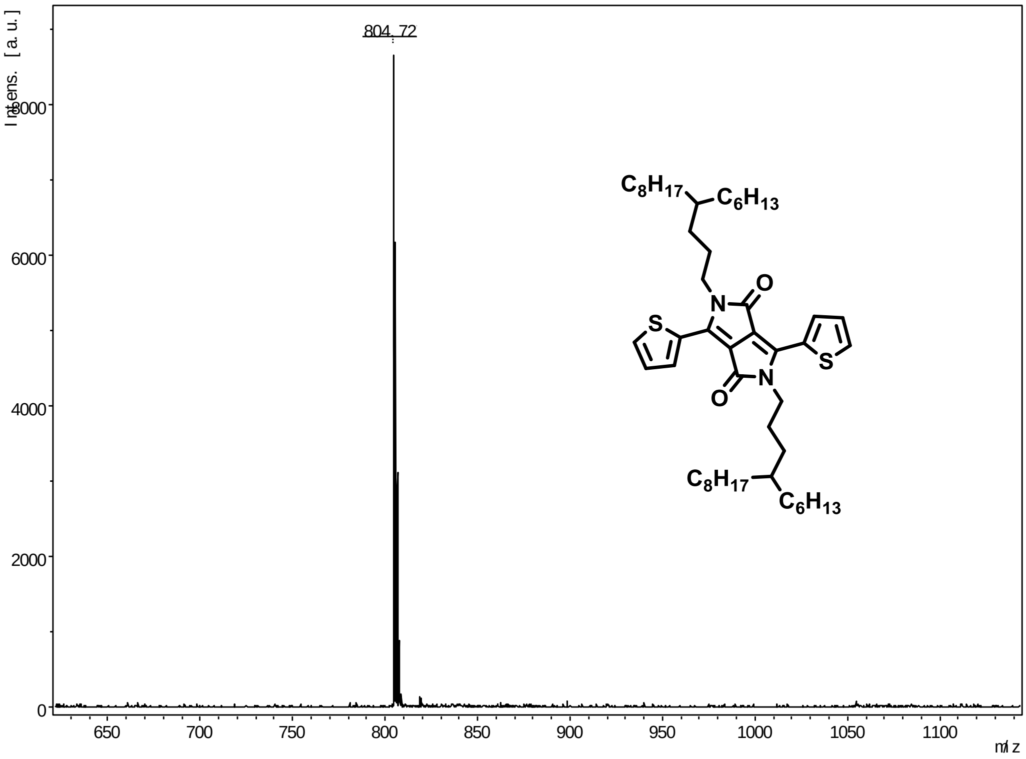

Figure S21. MALDI-TOF mass spectrum of **2**.

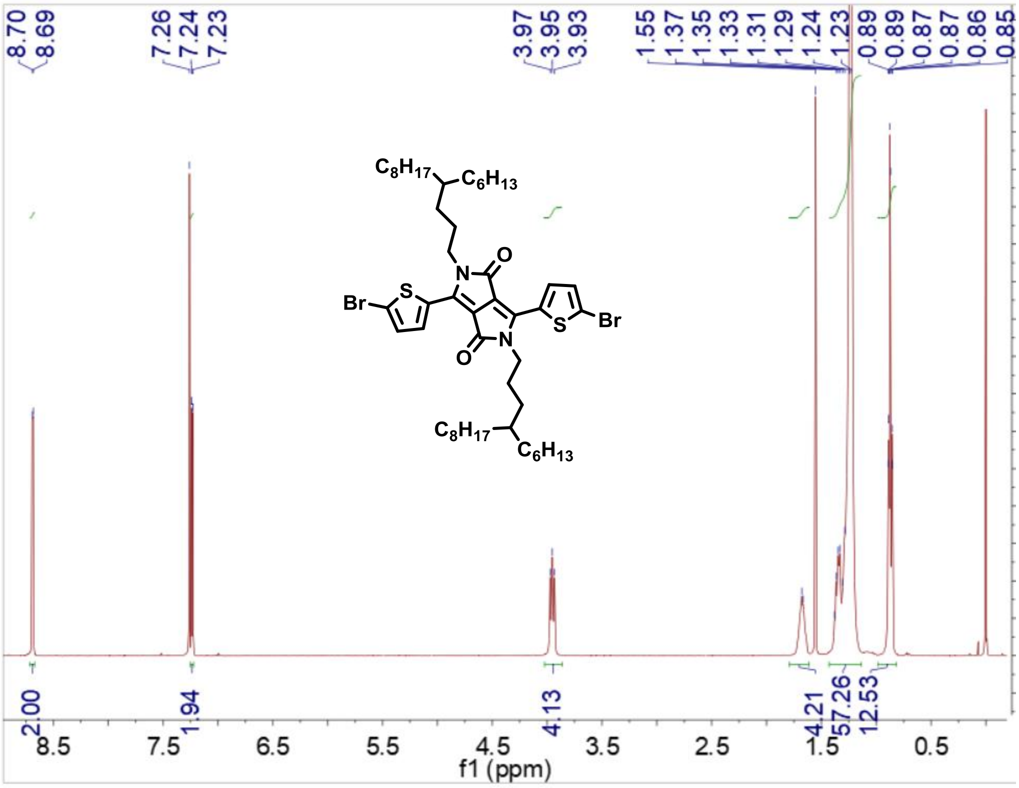

Figure S22. ^{1}H NMR spectrum (400 MHz, $CDCl_3$, 25 °C) of M2.

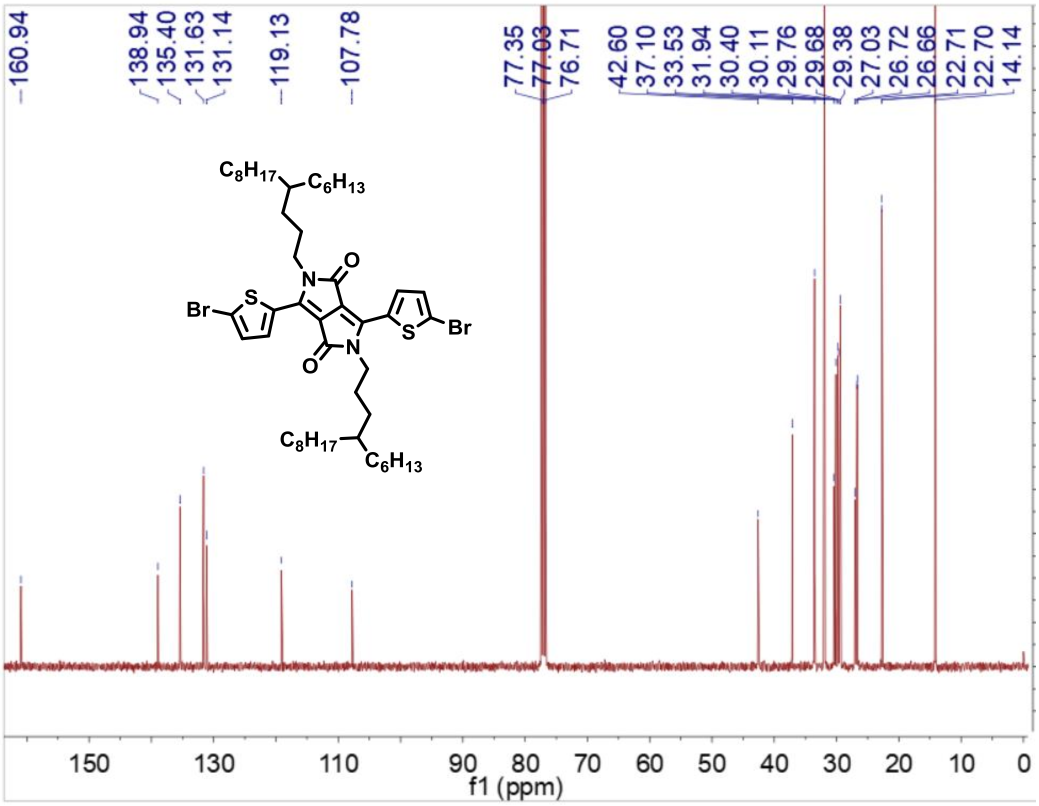

Figure S23. ^{13}C NMR spectrum (100 MHz, $CDCl_3$, 25 °C) of M2.

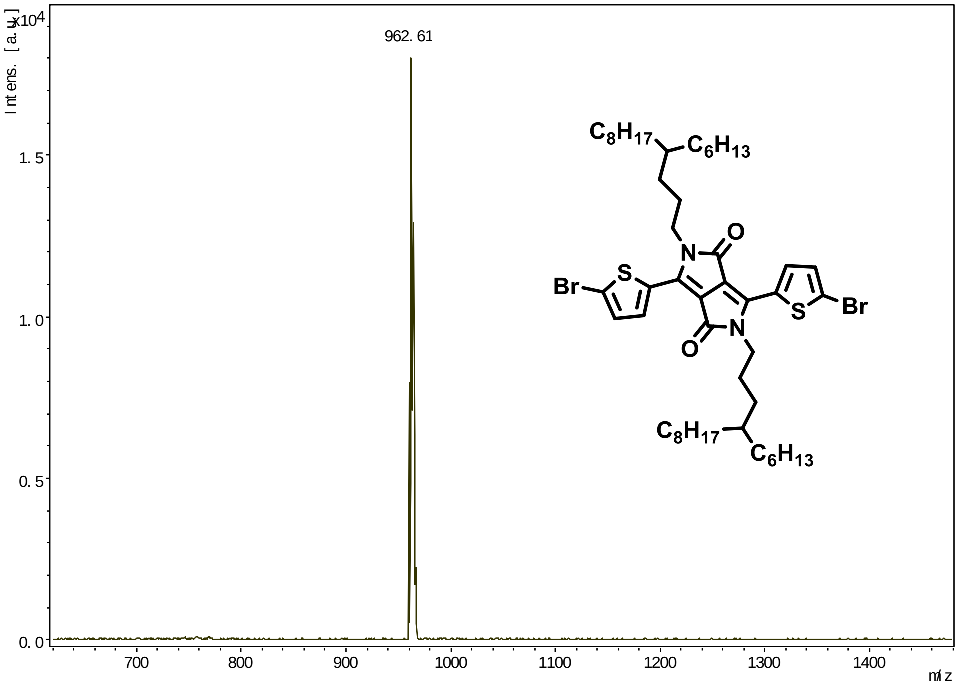

Figure S24. MALDI-TOF mass spectrum of M2.

5. References

[1] J. S. Lee, S. K. Son, S. Song, H. Kim, D. R. Lee, K. Kim, M. J. Ko, D. H. Choi, B. Kim, J. H. Cho, *Chem. Mater.* **2012**, *24*, 1316.

[2] J. Qi, X. Zhou, D. Yang, W. Qiao, D. Ma, Z. Y. Wang, *Adv. Funct. Mater.* **2014**, *24*, 7605.

[3] Y. Ji, C. Xiao, Q. Wang, J. Zhang, C. Li, Y. Wu, Z. Wei, X. Zhan, W. Hu, Z. Wang, R. A. J. Janssen, W. Li, *Adv. Mater*. **2016**, *28*, 943.

[4] H. H. Choi, K. Cho, C. D. Frisbie, H. Sirringhaus, V. Podzorov, *Nat. Mater*. **2017**, *17*, 2. [5] K. Lu, Y. Guo, Y. Liu, C. Di, T. Li, Z. Wei, G. Yu, C. Du, S. Ye, *Macromolecules*. **2009**,

42, 3222.

[6] J. Li, Q. Bao, C. M. Li, W. Zhang, C. Gong, M. B. Chan-Park, J. Qin, B. S. Ong, *Chem. Mater.* **2010**, *22*, 5747.

[7] M. Shao, Y. He, K. Hong, C. M. Rouleau, D. B. Geohegan, K. Xiao, *Polym. Chem*. **2013**, *4*, 5270.

[8] J. R. Matthews, W. Niu, A. Tandia, A. L. Wallace, J. Hu, W. Lee, G. Giri, S. C. B. Mannsfeld, Y. Xie, S. Cai, H. H. Fong, Z. Bao, and M. He, *Chem. Mater.* **2013**, *25*, 782.

[9] W. Lee, G. Giri, Y. Diao, C. J. Tassone, J. R. Matthews, M. L. Sorensen, S. C. B. Mannsfeld, W. Chen, H. H. Fong, J. B.-H. Tok, M. F. Toney, M. He, Z. Bao, *Adv. Funct. Mater.* **2014**, *24*, 3524.

[10] H. Yun, G. B. Lee, D. S. Chung, Y. Kim, S. Kwon, *Adv. Mater.* **2014**, *26*, 6612.

[11] J. Shaw, H. Zhong, C. P. Yau, A. Casey, E. Buchaca-Domingo, N. Stingelin, D. Sparrowe, W. Mitchell, M. Heeney, *Macromolecules.* **2014**, *47*, 8602.

[12] Y. Zhu, Z. Chen, Y. Yang, P. Cai, J. Chen, Y. Li, W. Yang, J. Peng, Y. Cao, *Org. Electron.* **2015**, *23*, 193.

[13] J. Cho, K. H. Cheon, K. H. Park, S. Kwon, Y. Kim, D. S. Chung, *Org. Electron.* **2015**, *24*, 160.

[14] J. Cho, K. H. Cheon, H. Ahn, K. H. Park, S. Kwon, Y. Kim, D. S. Chung, *Adv. Mater.* **2015**, *27*, 5587.

[15] K. H. Cheon, H. Ahn, J. Cho, H. Yun, B. T. Lim, D. J. Yun, H. Lee, S. Kwon, Y. Kim, D. S. Chung, *Adv. Funct. Mater.* **2015**, *25*, 4844.

[16] H. H. Choi, J. Y. Baek, E. Song, B. Kang, K. Cho, S. Kwon, Y. Kim, *Adv. Mater.* **2015**, *27*, 3626.

[17] J. Cho, K. H. Cheon, J. Ha, D. S. Chung, *Chem. Eng. J.* **2016**, *286*, 122.

[18] Y. Ji, C. Xiao, Q. Wang, J. Zhang, C. Li, Y. Wu, Z. Wei, X. Zhan, W. Hu, Z. Wang, R. A. J. Janssen, W. Li, *Adv. Mater*. **2016**, *28*, 943.

[19] G. Feng, Y. Xu, C. Xiao, J. Zhang, X. Zhang, C. Li, Z. Wei, W. Hu, Z. Wang, W. Li, *Polym. Chem.* **2016**, *7*, 164.

[20] B. Lim, H. Sun, J. Lee, Y. Noh, *Sci. Rep.* **2017**, *7*, 164.

[21] S. M. Lee, H. R. Lee, A. Han, J. Lee, J. H. Oh, C. Yang, *ACS Appl. Mater. Interfaces.* **2017**, *9*, 15652.

[22] Z. Fei, Y. Han, E. Gann, T. Hodsden, A. S. R. Chesman, C. R. McNeill, T. D. Anthopoulos, M. Heeney, *J. Am. Chem. Soc*. **2017**, *139*, 8552.

[23] M. Lee, M. J. Kim, S. Ro, S. Choi, S. Jin, H. D. Nguyen, J. Yang, K. Lee, D. U. Lim, E. Lee, M. S. Kang, J. Choi, J. H. Cho, B. Kim, *ACS Appl. Mater. Interfaces.* **2017**, *9*, 28817.

[24] T. L. Nguyen, C. Lee, H. Kim, Y. Kim, W. Lee, J. H. Oh, B. J. Kim, H. Y. Woo, *Macromolecules*. **2017**, *50*, 4415.

[25] Y. Wang, H. Tatsumi, R. Otsuka, T. Mori, T. Michinobu, *J. Mater. Chem. C,* **2018**, *6*, 5865

[26] H. Opoku, B. Nketia-Yawson, E. Shin, Y. Noh, *J. Mater. Chem. C,* **2018**, *6*, 661.

[27] S. Ding, Z. Ni, M. Hu, G. Qiu, J. Li, J. Ye, X. Zhang, F. Liu, H. Dong, W. Hu *Macromol. Rapid Commun.* **2018**, *39*, 1800225.

[28] G. N. Wang, F. Molina-Lopez, H. Zhang, J. Xu, H. Wu, J. Lopez, L. Shaw, J. Mun, Q. Zhang, S. Wang, A. Ehrlich, Z. Bao, *Macromolecules.* **2018**, *51*, 4976.

[29] S. Sanda, R. Nakamichi, T. Nagase, T. Kobayashi, K. Takimiya, Y. Sadamitsu, H. Naito, *Org. Electron.* **2019**, *69*, 181.

[30] R. Noriega, J. Rivnay, K. Vandewal, F. P. V. Koch, N. Stingelin, P. Smith, M. F. Toney, A. Salleo, *Nat. Mater.* **2013***, 12,* 1038.

[31] S. Himmelberger, K. Vandewal, Z. Fei, M. Heeney, A. Salleo, *Macromolecules*. **2014**, *47*, 7151.
